# Metabolic, fibrotic and splicing pathways are all altered in Emery-Dreifuss muscular dystrophy spectrum patients to differing degrees

**DOI:** 10.1093/hmg/ddac264

**Published:** 2022-10-25

**Authors:** Jose I de las Heras, Vanessa Todorow, Lejla Krečinić-Balić, Stefan Hintze, Rafal Czapiewski, Shaun Webb, Benedikt Schoser, Peter Meinke, Eric C Schirmer

**Affiliations:** Institute of Cell Biology, University of Edinburgh, Edinburgh, UK; Friedrich-Baur-Institute, Department of Neurology, LMU Clinic, Ludwig-Maximillians-University, Munich, Germany; Friedrich-Baur-Institute, Department of Neurology, LMU Clinic, Ludwig-Maximillians-University, Munich, Germany; Friedrich-Baur-Institute, Department of Neurology, LMU Clinic, Ludwig-Maximillians-University, Munich, Germany; Institute of Cell Biology, University of Edinburgh, Edinburgh, UK; Wellcome Centre for Cell Biology, University of Edinburgh, Edinburgh, UK; Friedrich-Baur-Institute, Department of Neurology, LMU Clinic, Ludwig-Maximillians-University, Munich, Germany; Friedrich-Baur-Institute, Department of Neurology, LMU Clinic, Ludwig-Maximillians-University, Munich, Germany; Institute of Cell Biology, University of Edinburgh, Edinburgh, UK

## Abstract

Emery-Dreifuss muscular dystrophy (EDMD) is a genetically and clinically variable disorder. Previous attempts to use gene expression changes to find its pathomechanism were unavailing, so we engaged a functional pathway analysis. RNA-Seq was performed on cells from 10 patients diagnosed with an EDMD spectrum disease with different mutations in seven genes. Upon comparing to controls, the pathway analysis revealed that multiple genes involved in fibrosis, metabolism, myogenic signaling and splicing were affected in all patients. Splice variant analysis revealed alterations of muscle-specific variants for several important muscle genes. Deeper analysis of metabolic pathways revealed a reduction in glycolytic and oxidative metabolism and reduced numbers of mitochondria across a larger set of 14 EDMD spectrum patients and 7 controls. Intriguingly, the gene expression signatures segregated the patients into three subgroups whose distinctions could potentially relate to differences in clinical presentation. Finally, differential expression analysis of miRNAs changing in the patients similarly highlighted fibrosis, metabolism and myogenic signaling pathways. This pathway approach revealed a transcriptome profile that can both be used as a template for establishing a biomarker panel for EDMD and direct further investigation into its pathomechanism. Furthermore, the segregation of specific gene changes into distinct groups that appear to correlate with clinical presentation may template development of prognostic biomarkers, though this will first require their testing in a wider set of patients with more clinical information.

## Introduction

Emery-Dreifuss muscular dystrophy (EDMD) is a genetically heterogeneous neuromuscular orphan spectrum disease affecting ~0.3–0.4 in 100 000 people (1), with clinical variability presenting even in family members carrying the same mutation (2–5). EDMD patients present typically in mid to late childhood with early contractures of elbows and Achilles’ tendons and progressive wasting of the lower leg and upper arm muscles. Cardiac involvement is also highly characteristic but tends to appear later in development and quite variably in time, though it tends to be reasonably uniform in the form it takes of cardiac conduction defects and dilated cardiomyopathy (6). Other features vary considerably in clinical presentation, leading to the usage of ‘Emery-Dreifuss-like syndromes’ (7,8): patients from the same pedigree can show remarkable phenotypic variation (2). The genetic variability is underscored by several confirmed linked genes and several additional candidate genes, although there are still some cases where no confirmed or candidate disease allele has been identified (9–11). The lack of large pedigrees in combination with its genetic heterogeneity, clinical variability, already some known modifier genes and limited patient numbers makes solving its pathomechanism difficult.

The original genes linked to EDMD, *EMD* encoding emerin and *LMNA* encoding lamin A/C, have both cytoskeletal and gene regulation roles leading to strong arguments for either function being responsible for the EDMD pathomechanism (12,13). The subsequent linking of nesprin and Sun proteins to EDMD (14,15) failed to lend clarity since they function in mechanosignal transduction (16). However, several recently linked genes have clear roles in genome organization and regulation (10), suggesting that this is the pathomechanism. These genes encode proteins that, such as emerin, are nuclear envelope transmembrane proteins (NETs) and seem to function by fine-tuning muscle gene expression by promoting the release of pro-myogenic genes from the nuclear periphery to enhance their activation while concomitantly recruiting metabolism genes (many from the alternative differentiation pathway of adipogenesis) to the nuclear envelope to better repress them (17–19). EDMD mutations were found in five muscle-specific NETs with this genome organization function, PLPP7 (also known as NET39), WFS1, TMEM38A, TMEM201 and TMEM214, and each tested had some specificity in the sets of genes that they target, though there was also some overlap (17). These studies together with the wide range of lamin and emerin gene regulatory activities led us to the non-traditional hypothesis for the EDMD pathomechanism whereby moderate reductions in many genes could yield the same phenotype as shutting down a central gene of a particular pathway. Accordingly, we considered that searching for uniformity among patients in altered pathways might be more revealing than searching for uniformity in expression changes of particular genes.

The only previous study, to our knowledge, using gene expression changes to identify critical misregulated genes underlying EDMD pathophysiology, focused on the identification of genes altered specifically in EDMD compared with a set of 10 other muscular dystrophies (20). This study only considered eight total *LMNA*- or *EMD*-linked cases of EDMD, but EDMD now has many more genes and modifiers linked to it and, moreover, there is a wider clinical spectrum of EDMD-like phenotypes (11). Their analysis indicated potential abnormalities in the regulation of cell cycle and myogenic differentiation, associated with perturbations in the pRB/MYOD/LMNA hub, which were consistent with changes in an *Emd^−/−^* mouse model (21). Roughly a fifth each of EDMD mutations occurs in *LMNA* and *EMD* while another 5–6% are collectively caused by four other widely expressed nuclear envelope proteins nesprin 1 (encoded by *SYNE1*), nesprin 2 (encoded by *SYNE2*), Sun1 (encoded by *SUN1*) and FHL1 (encoded by *FHL1*) (14,15,22–24)). Another approximately 20% of EDMD mutations were accounted for by muscle-specific NETs that regulate muscle-specific genome organization (10). These include NET39 (encoded by *PLPP7*), TMEM38A (encoded by *TMEM38A*), WFS1 (encoded by *WFS1*), NET5 (encoded by *TMEM201*) and TMEM214 (encoded by *TMEM214*) that affect 3D gene positioning with corresponding effects on expression (17,19). Accordingly, we sought to search for commonly affected pathways from a much wider range of EDMD-linked genes including *LMNA*, *EMD*, *FHL1*, *SUN1*, *SYNE1*, *PLPP7* and *TMEM214* alleles on the expectation that, covering an even wider genetic and clinical spectrum, the most important pathways for EDMD pathophysiology would be highlighted.

## Results

### RNA-Seq analysis of EDMD patient cells

**Figure 1:**
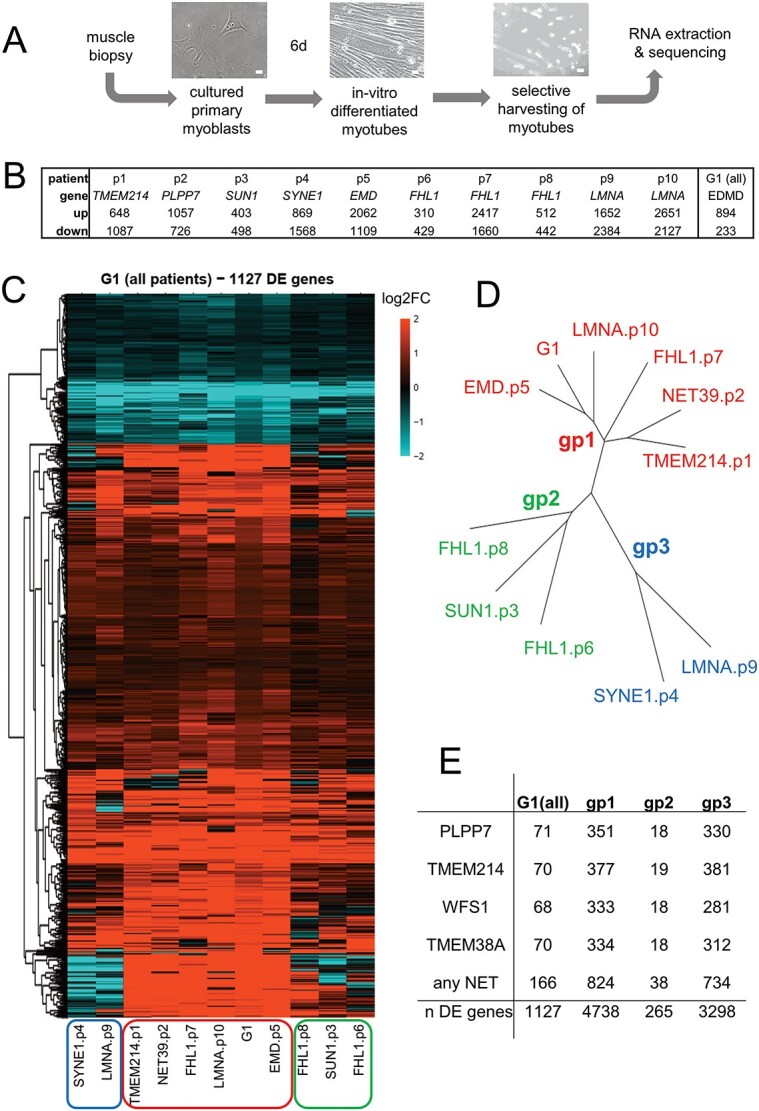
RNA-Seq for EDMD. (**A**) Workflow. Muscle biopsies were taken from the regions described in Table 1 and myoblasts recovered. These were then differentiated *in vitro* into myotubes, and myotubes selectively recovered by partial trypsinization. Myotube RNA was extracted and used for sequencing. Scale bar 20 μm. (**B**) Number of genes differentially expressed (FDR 5%) for each individual patient and for all patients considered together as a single group (denoted as ‘G1’). (**C**) Heatmap of log2FC values for genes changing expression in EDMD patients compared with healthy controls (G1). Red is upregulated and blue is downregulated. Black indicates no change. The G1 lane is the averaged data across all patients. (**D**) Dendrogram showing the relationships between patients, which fall into three broad groups. (**E**) Overlaps with genome-organizing NETs gene targets. The number of genes altered by knockdown of muscle-specific genome-organizing NETs Wfs1, Tmem38a, NET39 or Tmem214 that were also altered in the EDMD patient cells is given. G1 refers to the analysis of all 10 patients as a single group against the healthy controls, while gp1–3 refers to the three subgroups identified. Total numbers of differentially expressed (DE) genes are given for each.

We performed RNA-Seq on myotubes differentiated *in vitro* from myoblasts isolated from 10 unrelated clinically diagnosed EDMD patients with distinct mutations in seven different genes to sample the genetic diversity of EDMD (Fig. 1). Patient mutations were TMEM214 p.R179H, PLPP7/NET39 p.M92K, Sun1 p.G68D/G388S, Nesprin 1 p.S6869^*^, Emerin p.S58Sfs^*^1, FHL1 mutations c.688 + 1G > A, p.C224W and p.V280M, Lamin A/C mutation p.T528K and Lamin A mutation p.R571S (this last mutation occurs in an exon absent from the lamin C splice variant). These mutations covered a wide range of clinical phenotypes with the age of onset ranging from early childhood to adult life and associated pathology ranging from no reported contractures to rigid spine (Table 1). Myoblast isolation followed by *in vitro* differentiation was chosen over isolating mRNA directly from the tissue samples in order to try to capture the earliest changes in gene expression due to the disease mutations *i.e.* the most likely to initiate the pathomechanism. Differentiating isolated myoblasts cells also reduces tertiary effects and variation from the age of the patients, range in time from onset to when biopsies were taken and differences in biopsy site (Table 1). These patient variables could also affect the efficiency of myotube differentiation; so, to ensure that different percentages of undifferentiated cells in the population did not impact measuring gene expression changes, the myotubes were specifically isolated by short trypsinization thus removing all myoblast contamination (Fig. 1A and Supplementary Material, Fig. S1). To define the baseline for comparison, two age-matched healthy controls were similarly analyzed. Samples from all patients and controls all yielded high-quality reads ranging between 56 and 94 million paired-end reads (Supplementary Material, Table S1).

**Table 1:** Patients and controls used in this study. List of patients and controls including gene, mutation, age at biopsy, age of onset, sex, creatine kinase (CK) levels, clinical features and the muscle groups from whence biopsies were obtained. Note that the normal range for CK levels is 25–200 U/l.

ID	Gene	Mutation	RNAseq	Metabolic analysis	Age	Age of onset	sex	CK(U/l)	Muscle weakness	Contractures	Cardiac defects	Family history	Neurological defects	Biopsy location	Other clinical information
C1	Control		Yes	Yes	35		M		No	No	No			Gastrocnemius caput laterale	
C2	Control		Yes	Yes	13		M		No	No	No				
C3	Control			Yes	43		M		No	No	No			Biceps brachii	
C4	Control			Yes	36		F		No	No	No			Biceps brachii	
C5	Control			Yes	49		F		No	No	No			Vastus lateralis	
C6	Control			Yes	49		F		No	No	No				
C7	Control			Yes	53		M		No	No	No			Vastus lateralis	
P1	TMEM214	p.179H	Yes	Yes	48	28	F	350	Limb girdle weakness	No	No	Sister; father pacemaker		Rectus femoris	LGMD, ptosis, type 2 fiber atrophy, Trendelenburg sign, Gowers sign
P2	PLPP7	p.M92K	Yes	Yes	34	16	F	400	Distal myopathy pattern	Yes	No	No		Vastus lateralis	Distal myopathy, Trendelenburgh sign calf atrophy, tibial muscle more affected, Achilles contracture
P3	SUN1	p.G68D p.G388S	Yes	Yes	9	10	M	>3000	Yes	Yes	Yes			Quadriceps femoris	
P4	SYNE1	p.6869^*^ p.6869^*^	Yes	Yes	15		F	347	Yes				yes	Quadriceps femoris	Growth deficit, ataxia
P5	EMD	p.S58Sfs^*^1	Yes	Yes	17	12	F	278	Limb girdle weakness	Yes	Yes	Father, uncle and grandmother	no	Biceps brachii	Scapula alata scoliosis, mild proximal paresis, calf hypertrophy, Achilles contracture
P6	FHL1	c.688 + 1G > A	Yes		28		M	1000	Limb girdle weakness	Yes	Yes	Mother and cousin	no	Biceps brachii	Moderate proximal weakness, neck flexor contracture, Achilles tendon axial atrophy
P7	FHL1	p.C224W	Yes	Yes	46		M	300–1000	Limb girdle weakness	Yes	Yes	Mother and brother	no	Tibialis anterior	Moderate proximal weakness, neck flexor contracture, Achilles tendon axial atrophy
P8	FHL1	p.V280M	Yes	Yes	21		M	231	Limb girdle weakness	Yes	Yes	Brother	no	Gastrocnemius caput laterale	Rigid spine, moderate proximal weakness, neck flexor contracture, axial atrophy
P9	LMNA	p.T528K	Yes		2		M		Yes				no		
P10	LMNA	p.R571S	Yes	Yes	24	9	F	up to 12 000	Limb girdle weakness	No	No	No	no	Vastus lateralis	Degenerative myopathy, mild proximal weakness
P11	LMNA	p.R545C		Yes	18		M		Yes	Yes	Yes				
P12	LMNA	p.R543W		Yes	12		F		Yes					Vastus lateralis	Acrogeria
P13	Unknown	Unknown		Yes	14		M	2800	Yes	Yes	Yes			Tibialis anterior	Degenerative myopathy
P14	FHL1	p.C224W		Yes	42		M	1700	Yes	Yes	Yes			Vastus lateralis	Myofibrillar myopathy

First, we compared each individual patient against the controls. Compared with the controls, each individual patient had between 310 and 2651 upregulated genes and between 429 and 2384 downregulated genes with a false discovery rate (FDR) of 5% (Fig. 1B). The large difference in the number of differentially expressed (DE) genes between patients suggested large heterogeneity. When we calculated the intersection of DE genes in all patients, only three genes were similarly downregulated (*MTCO1P12*, *HLA-H*, *HLA-C)*, and one upregulated (*MYH14)* at 5% FDR, indicating a high degree of variation between patients (Supplementary Material, Fig. S2A). *MTCO1P12* is a mitochondrially encoded pseudogene that has been reported to be severely downregulated in inflammatory bowel disease, associated with reduced mitochondrial energy production (25). HLA-C is a member of the MHC class I and is involved in interferon gamma signaling, while HLA-H is a pseudogene derived from HLA-A which may function in autophagy (26–28). Mutations in non-muscle myosin gene *MYH14* appear to be associated with hearing loss rather than muscle defects (29,30), although it has also been recently linked to mitochondrial fission defects (31).

The small number of intersecting genes was expected, given the heterogeneity and the number of samples. However, this kind of comparison underestimates the underlying similarity since all it takes is one patient for any given gene to miss the arbitrary 5% FDR cutoff and the gene would not be selected. Because the goal of this study is to identify common features among a sample of EDMD spectrum patients, instead of focusing on individual patients we next compared all patients together as a single group against the controls, and we denoted this analysis as G1 (one single group) throughout the text (Fig. 1). The G1 analysis revealed a set of 1127 DE genes (894 upregulated and 233 downregulated) with a 5% FDR cutoff. Preliminary examination of this set of genes across the 10 patients suggested that they might fall into two or three broadly distinct profiles with a main group comprising half of the patients that includes the patient with the classical emerin mutation. Around 60% of the genes were altered in the same direction in all 10 patients (Fig. 1 and Supplementary Material, Fig. S2B and C). Thus, while the majority of the 1127 genes behave in a similar fashion (albeit with differences in gene expression levels), there are substantial underlying differences and apparent subgroups of patients with more similar expression profiles that might reflect clinical variation in EDMD.

Hierarchical clustering identified one main subgroup comprising half of the patients that includes the EMD mutation, with the remaining patients falling more loosely into two smaller groups (Fig. 1C and D**)**. While it is tempting to suggest these groups may represent separate EDMD spectrum subcategories, the small cohort employed precludes this conclusion. However, we reasoned that the distinction may bear clinical relevance and provide proof of principle that transcriptome analysis could assist clinicians in diagnosis and prognosis of EDMD spectrum patients. The three groupings were: group 1—Emerin p.S58Sfs^*^1, Tmem214 p.R179H, NET39 p.M92K, Lamin A p.R571S, FHL1 p.C224W; group 2—Sun1 p.G68D/G388S, FHL1 c.688 + 1G > A, FHL1 p.V280M; group 3—Nesprin 1 p.S6869^*^, Lamin A/C p.T528K (Fig. 1D). The same groups were independently identified using a principal component analysis (PCA) (Supplementary Material, Fig. S2D). Of particular note, both PCA and t-distributed stochastic neighbor embedding analyses revealed that clustering was independent of parameters such as patient gender or age or myotube enrichment differences (Supplementary Material, Fig. S3). Moreover, the several FHL1 and lamin mutations tested segregated into different expression subgroups. At the same time, the more recently identified EDMD mutations in TMEM214 and NET39 segregated with more classic emerin, FHL1 and lamin A mutations, further indicating the likelihood that their genome organizing functions could mediate core EDMD pathophysiology.

The hypothesis that EDMD is a disease of genome organization misregulation is underscored by the fact that 15% of the genes changing expression in EDMD patient cells were altered by knockdown of at least one of the four muscle-specific genome-organizing NETs that we previously tested (17) (Fig. 1E). Interestingly, in that study most of the genes altered by knockdown of NET39, TMEM38A and WFS1 were non-overlapping, while those altered by knockdown of TMEM214 exhibited considerable overlap with the sets altered by each of the other NETs (17). However, here there were roughly 70 DE genes overlapping with the sets of genes altered by knockdown of each individual NET while the total number of DE genes under the regulation of any of the four NETs was 165, indicating an enrichment in the EDMD DE set for genes influenced by multiple NETs (Fig. 1E). Thus, it is not surprising that NET39 and TMEM214 were both segregated together. Another interesting observation is that the number of NET-regulated genes overlapping with group 1 and group 3 was similar, but much fewer were overlapping for group 2. This suggests that gene misregulation in groups 1 and 3 might be more strongly mediated by the muscle-specific NET-gene tethering complexes than in group 2.

### Functional pathway analysis of gene expression changes in EDMD patient cells

The primary aims of this study were to determine whether a functional pathway analysis would be more effective at revealing the likely underlying EDMD pathomechanism than just looking for uniformly altered genes and, if so, to identify candidate biomarkers from the affected pathways, though these would require subsequent validation due to the limited number of patient cells available for analysis. Before using this approach with our wider set of EDMD alleles, we applied a pathway analysis to the data from the previous microarray study by Bakay and colleagues where just *LMNA* and *EMD* mutations were considered (20). We reanalyzed Bakay’s EDMD data and extracted the subset of DE genes with FDR of 5% (1349 and 1452 upregulated and downregulated genes, respectively). In order to identify enriched functional categories within each set of DE genes, we used g:Profiler (32). This tool calculates the expected number of genes to be identified for any given functional category by chance and compares it to the number of genes observed. We selected categories that were significantly enriched with an FDR of 5%. The resulting list was then summarized by selecting representative classes using a similar approach to Revigo (33), but extended to other functional category databases in addition to gene ontology (GO) terms. Briefly, similarity matrices were generated by calculating pairwise Jaccard similarity indices between categories and applying hierarchical clustering to group together similar functional categories based on the genes identified. Redundancy was then reduced by choosing a representative category from each group.

**Figure 2:**
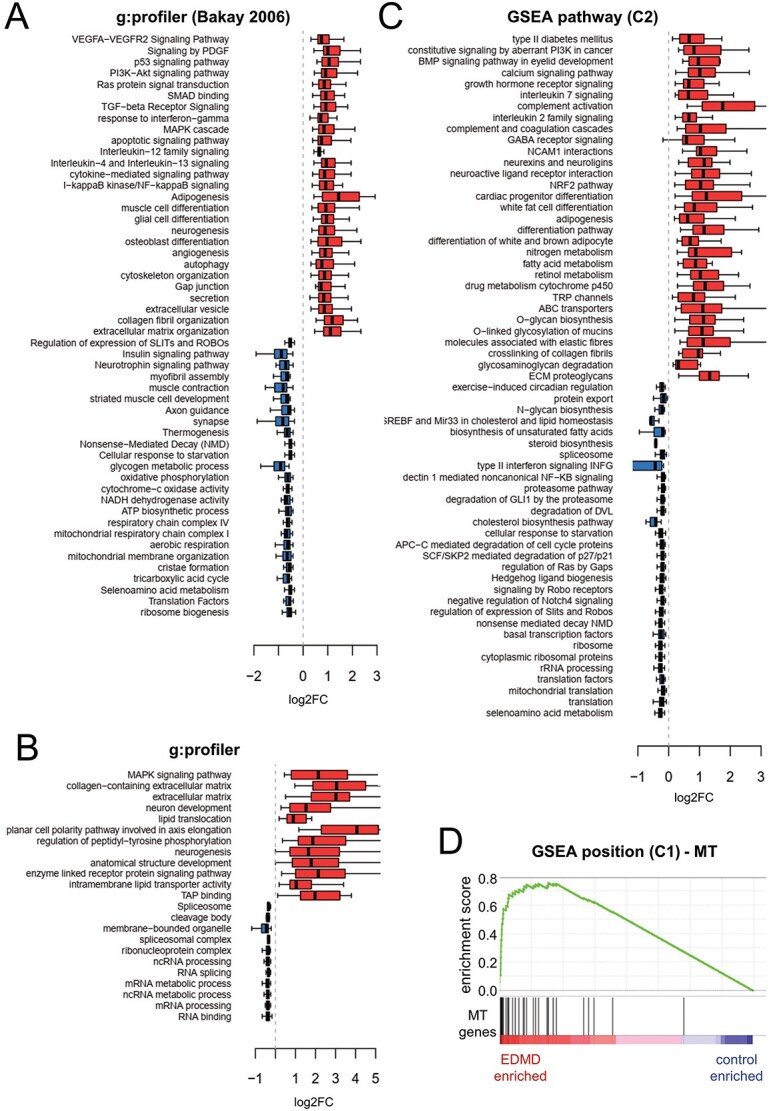
Search for functional categories changing in EDMD patients. (**A**) Box plot of log2FC values for differentially expressed genes within significantly enriched functional categories in EDMD patients analyzed in the Bakay study using g:profiler. (**B**) Box plot of log2FC values for differentially expressed genes within significantly enriched functional categories in EDMD patients analyzed in this study using g:profiler. (**C**) Box plot of log2FC values for leading edge genes within significantly enriched functional categories in EDMD patients analyzed in this study using GSEA pathway (C2 geneset collection: canonical pathways). (**D**) GSEA enrichment plot for mitochondrially encoded genes. GSEA analysis using the C1 geneset (positional) revealed an upregulation of mitochondrially encoded genes. See Supplementary Material, Table S3 for further details.

The functional categories enriched in the set of EDMD-upregulated genes revealed defects in cytokine signaling, organization of the extracellular matrix (ECM), and various signaling pathways important for muscle differentiation and function (*e.g.* PI3K-Akt, TGF-beta, SMADs). In addition, there was an aberrant upregulation of alternative differentiation pathways, notably adipogenesis but also angiogenesis and osteogenesis. The functions highlighted among the downregulated genes were largely related to metabolism, mitochondrial especially, as well as ribosome biogenesis, muscle contraction and myofibril assembly (Fig. 2A). Applying the same methodology to our wider set of patient alleles highlighted fewer pathways than what we observed in the Bakay EDMD data, an expected outcome for the hypothesis that sampling the wider genetic variation in the disorder might hone in on the most central pathways for the pathomechanism. Among the upregulated categories, neurogenesis and ECM-related functions stood out, as well as MAPK signaling, lipid transport and TAP binding which are linked to interferon-gamma signaling (34). One category stands out among the downregulated genes: RNA splicing (Fig. 2B). The data above used g:Profiler which is very sensitive to the number of DE genes identified because it looks for statistical overrepresentation of genes belonging to specific functional categories among a set of previously identified DE genes. By contrast, gene set enrichment analysis (GSEA) (35) does not prefilter the data and instead ranks all genes according to the difference in expression between the two conditions tested: controls and EDMD. Next, it determines whether the distribution in the ranked list for any given functional category is random or significantly enriched statistically at either end of the ranked list. This method is especially sensitive for detecting functional categories where many genes are altered by a small amount and does not consider individual gene *P*-values. Therefore, we also applied GSEA to our data, querying several functional genesets within the Reactome, KEGG and WikiPathways databases, found in the Molecular Signatures Database (MSigDB) (36). This approach identified a larger set of functional categories that generally expanded on those identified by g:Profiler and matched better what we observed from the Bakay EDMD geneset, with strong links to ECM organization and cytokine signaling that may be relevant to fibrosis, differentiation, metabolism and splicing (Fig. 2C).

**Figure 3:**
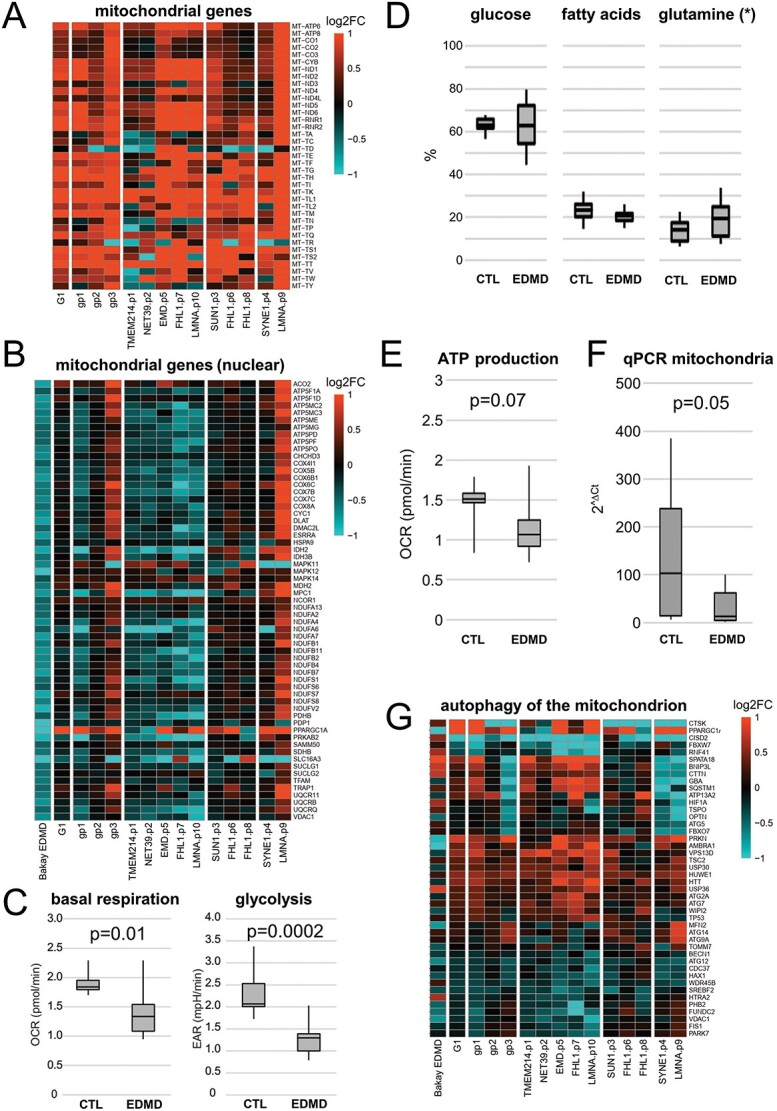
Metabolism changes confirmed in patients. (**A**) Heatmap of all mitochondria-encoded genes showing almost uniform upregulation in EDMD patients. (**B**) Heatmap of nuclear-encoded downregulated genes in Bakay’s study that supported mitochondria function. Downregulation is also generally observed in patients belonging to group 1, but not group 2 or 3. Functional analyses performed in primary patient (*n* = 12) and control (*n* = 7) myoblast cultures for (**C**) glycolysis and basal respiration show a significant reduction of both in EDMD myoblast cultures. (**D**) Testing for fuel dependency we could not observe any changes (^*^ glucose and fatty acids have been measured; glutamine calculated). (**E**) ATP production is reduced in EDMD samples. All functional experiments have been repeated in at least three independent experiments. (**F**) qPCR of mitochondria shows a reduction of mitochondria in EDMD myoblasts. (**G**) Heatmap of mitophagy genes (GO-BP, GO:0000422, Autophagy of the mitochondrion) that are differentially expressed in at least one of Bakay’s EDMD, G1 analysis (all 10 patients analyzed as a single group) or G3 analysis (each of the three subgroups analyzed separately).

An expansion of categories for metabolic functions included specific categories for diabetes mellitus, adipogenesis, white and brown fat differentiation, nitrogen metabolism fatty acid metabolism, retinol metabolism and many others. Similarly, there was an expansion of cytokines supporting inflammation for the fibrotic pathways and proteoglycans and elastin adding to the previous emphasis on collagens for ECM defects. Among the differences between our data and Bakay’s EDMD data, two categories stand out: RNA splicing and calcium signaling, which were only observed in our data. It is unclear how much this reflects using terminally differentiated muscle material versus early stages of differentiation *in vitro*, or a factor of microarray versus RNAseq analysis. In some cases, this is most likely due to the different transcriptome platform used. For example, applying GSEA to genomic positional genesets revealed near uniform upregulation of all mitochondrially encoded genes (Figs 2D and 3A). This could not be observed on Bakay’s data because the microarrays did not contain probes for mitochondrially encoded genes. The upregulation of mitochondrial transcripts could lead to increased oxidative stress (37). This finding provides yet another mechanism that could lead to metabolic dysregulation on top of the alterations already indicated by the nuclear genome transcript changes. This further underscored the need to test for actual metabolic deficits in the patient cells themselves as well as to further investigate the other functional pathways highlighted by this analysis.

### Detailed analysis of metabolic pathways uniformly altered in EDMD patients

Since metabolic disruption has been previously reported to affect muscle differentiation/myoblast fusion (38), we decided to investigate this further. While we identified a general upregulation of mitochondrially encoded genes, Bakay’s data showed a downregulation of several classes related to mitochondrial function (Fig. 2A) which was due entirely to nuclear-encoded genes, as there were no mitochondrial genes represented in the microarrays. When we checked the behavior of those genes in our data, we did not observe the same downregulation when considering all 10 patients as a single group (Fig. 3B). However, this is largely due to variability among the patient subgroups identified earlier, suggesting a mechanistic breakdown between them. Group 1 which contained half of our patients, including emerin and lamin A mutations, exhibited the same general downregulation of the nuclear-encoded mitochondrial genes. In contrast, group 2 displayed no alteration in gene expression, while group 3 showed upregulation although this was driven mostly by patient 9 (*LMNA*) with the other patient in the group, patient 4 (*SYNE1*), displaying very few changes. While no single gene was uniformly altered in the same direction for all patients, several genes from glycolytic and oxidative metabolism pathways, typically encoding components of mitochondrial complexes, were altered in all tested patients. Other non-mitochondrial metabolic pathways were also altered such as lipid translocation (Fig. 2C and Supplementary Material, Table S3). Interestingly, downregulation of nuclear-encoded mitochondrial genes was also generally observed in other muscular dystrophies included in the study by Bakay and colleagues (Supplementary Material, Fig. S4).

To investigate the relevance of these gene changes to cellular metabolism, we performed real-time metabolic analysis using the Seahorse XFp Extracellular Flux Analyzer. Myoblasts isolated from the above patients plus several additional EDMD patients and controls were tested, so that we had a total of 14 EDMD patients and 8 controls for this analysis (Table 1). Probing for glycolysis, a significant reduction of the extracellular acidification rate in the EDMD samples was observed (Fig. 3C). Next, we investigated mitochondrial function. When testing for basal respiration there was also a significant reduction of the oxygen consumption rate in the EDMD samples (Fig. 3C). There were no significant differences in fuel dependency, but ATP production was considerably reduced in the EDMD samples (Fig. 3D and E). The significant reduction in mitochondrial respiration raised another possibility to investigate that the absolute number of mitochondria might also be down due to problems in mitochondria biogenesis. Therefore, we quantified relative mitochondria numbers by qPCR. This revealed a clear reduction in mitochondria numbers (Fig. 3F), which with the generally elevated mitochondrial genome transcripts would suggest that a reduction in mitochondria numbers resulted in an overcompensation of expression which in turn could have resulted in inhibiting mitochondrial fission and repair. Thus, we also investigated whether genes in pathways associated with mitophagy were altered in the patients. Indeed, multiple mitophagy pathway genes were altered in all patients (Fig. 3G). Although no one individual gene was altered in all the patients, it is worth noting *CISD2* is significantly downregulated in most patients. Reduction of CISD2 has been linked to degeneration of skeletal muscles, misregulated Ca^2+^ homeostasis and abnormalities in mitochondrial morphology in mouse (39), as well as cardiac dysfunction in humans (40).

### Detailed analysis of other pathways uniformly altered in EDMD patients

**Figure 4:**
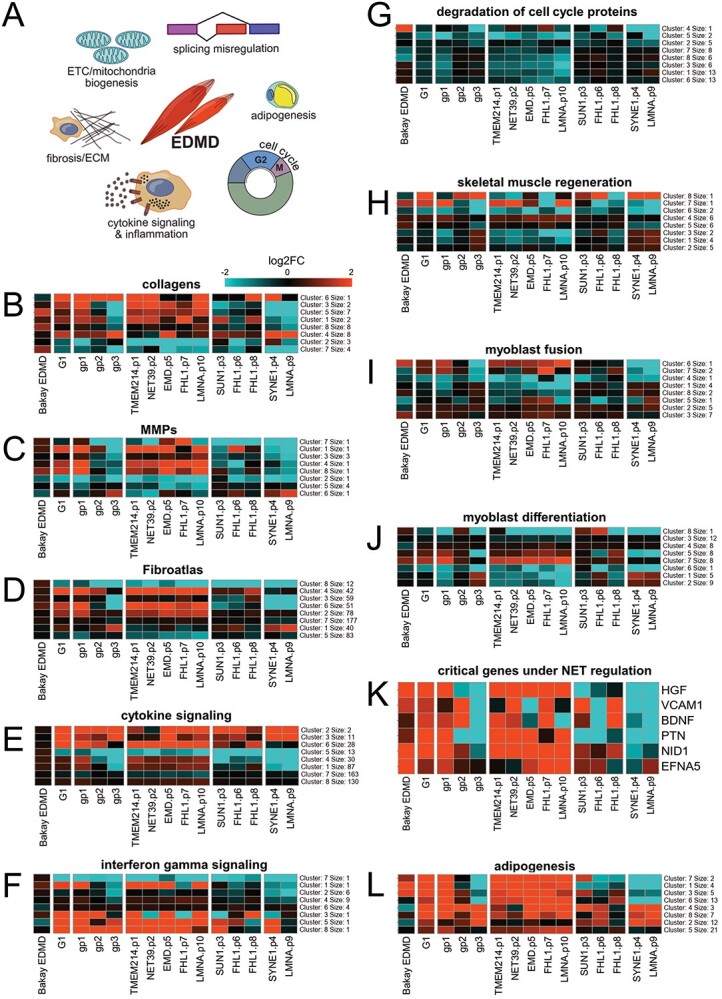
Genes changing expression within functional groups supporting ECM/fibrosis/myotube fusion. (**A**) Summary of altered functions in EDMD. (**B**) Heatmap showing gene expression changes for collagens. (**C**) Heatmap showing gene expression changes for matrix metalloproteinases. (**D**) Heatmap showing gene expression changes for general proteins involved in fibrosis according to FibroAtlas (http://biokb.ncpsb.org.cn/fibroatlas). (**E**) Heatmap showing gene expression changes for cytokine signaling proteins (GO-BP, GO:0019221). (**F**) Heatmap showing gene expression changes for interferon-gamma signaling proteins (WikiPathways, WP619). (**G**) Heatmap showing downregulation of genes involved in the degradation of cell cycle proteins. GSEA identified downregulation in EDMD patients of REACTOME hsa-1 741 423 (APC-C mediated degradation of cell cycle proteins) and hsa-187 577 (SCF/SKP2 mediated degradation of p27/p21) (Fig. 2C). Both genesets were combined. (**H**) Heatmap showing gene expression changes for skeletal muscle regeneration proteins (GO-BP, GO:0043403). (I) Heatmap showing gene expression changes for myoblast fusion (GO-BP, GO:0007520). (**J**) Heatmap showing gene expression changes for myoblast differentiation (GO-BP, GO:0045445). (**K**) Heatmap showing gene expression changes for specific genes functioning in myotube fusion that are under genome-organizing NET regulation. (**L**) Heatmap showing the upregulation of many pro-adipogenic genes (Wikipathways, WP236). All heatmaps were generated from differentially expressed genes in at least one of Bakay’s EDMD, G1 analysis (all 10 patients analyzed as a single group) or G3 analysis (each of the three subgroups analyzed separately). K-means unsupervised hierarchical clustering was used to summarize the heatmap into eight clusters of roughly coexpressing genes. Full heatmaps displaying gene names are provided in Supplementary Material, Figures S5–S11. The number of genes per cluster is indicated on the right of each heatmap. Red and blue indicate upregulation and downregulation, respectively.

Several studies suggest that the timing of several aspects of myotube fusion could underlie some of the aberrancies observed in patient muscle (41) and, though it is unclear whether fibrosis drives the pathology or is a consequence of the pathology, fibrosis has been generally observed in EDMD patient biopsies. Contributing to these processes could be several subpathways that fall variously under the larger pathways for ECM/fibrosis, cell cycle regulation and signaling/differentiation (Fig. 4A). As for the metabolic analysis, no individual genes were altered in cells from all patients, but every patient had some genes altered that could affect ECM through changes in collagen deposition (Fig. 4B). For example, 35 out of 46 collagen genes exhibited changes in at least one comparison (Bakay EDMD, G1 or one of the subgroups gp1, gp2 and gp3) and all patients had multiple of these genes altered (Supplementary Material, Fig. S5). Note that it often appears visually that the Bakay data in the first column has little change when viewing the cluster analysis, but when looking at the full set of genes listed in the matching supplemental figures there are definitely some genes strongly changing, just not necessarily the same ones. This may be due to differences in the myogenic state of the material studied: while Bakay and colleagues used muscle biopsies containing terminally differentiated muscle fibers, we focused on the earlier stages of myogenesis by *in vitro* differentiating cultured myoblasts obtained from muscle biopsies. Despite this, it is important to note that while different genes may be affected, most of the same pathways were highlighted in both Bakay’s and our study. Collagens COL6A1, COL6A2, COL6A3 and COL12A1 are linked to Bethlem muscular dystrophy (42–45) and, interestingly, all these collagens were upregulated in group 1 patient cells and downregulated in group 3 patient cells (Supplementary Material, Fig. S5). Matrix metalloproteinases, which participate in the degradation and remodeling of the ECM, were also altered with 13 out of 28 matrix metalloproteinases exhibiting changes in at least one of the comparisons and all patients had multiple of these genes altered (Fig. 4C and Supplementary Material, Fig. S5). Notably the metalloproteinase MMP1 (collagenase I), which has been proposed to resolve fibrotic tissue (46), was downregulated in all but one patient, as well as in Bakay EDMD samples. Likewise, multiple genes associated with fibrosis from FibroAtlas (Fig. 4D and Supplementary Material, Fig. S6) and with inflammation that would support fibrosis such as cytokine (Fig. 4E and Supplementary Material, Fig. S7) and INF-gamma signaling (Fig. 4F and Supplementary Material, Fig. S8) were affected in all patients. In fact, out of 941 genes in FibroAtlas there were 542 altered between all the patients. Heatmaps of gene clusters with similar expression patterns are shown in Figure 4, but more detailed individual panels with all gene names listed are shown in Supplementary Material, Figures S5–11. A few genes that stand out for their functions within the INF-gamma signaling pathway include IRF4 that is a regulator of exercise capacity through the PTG/glycogen pathway (47) and ILB1 that helps maintain muscle glucose homeostasis (48) such that both could also feed into the metabolic pathways altered.

Another subpathway critical for myogenesis and the timing and integrity of myotube fusion is cell cycle regulation. Cell cycle defects could lead to spontaneous differentiation and were previously reported in myoblasts from EDMD patients and in tissue culture cell lines expressing emerin carrying EDMD mutations which could lead to depletion of the stem cell population (41,49). All tested EDMD patients exhibited downregulation of multiple genes involved in the degradation of cell cycle proteins (Figs 2C and 4G, and Supplementary Material, Fig. S8) which could indicate an uncoupling of the joint regulation of cell cycle and myogenesis program (50), for example cells starting to fuse when they should still be dividing or *vice versa*.

Other pathways in addition to ECM deposition directly associated with myogenic differentiation, myoblast fusion and muscle regeneration were also altered in all patients (Fig. 4H–J and Supplementary Material, Figs S9 and S10), though, again, no single gene in these pathways was altered in the same way in all patients’ cells. Poor differentiation and myotubes with nuclear clustering were observed in differentiated EDMD myoblast cultures (14) and in the mouse C2C12 differentiation system when EDMD-linked NETs were knocked down (17).

Previous work using C2C12 cells identified six genes whose products are required in the early differentiation stages and were under the regulation of muscle-specific genome-organizing NETs (17). These genes (*NID1*, *VCAM1*, *PTN*, *HGF*, *EFNA5* and *BDNF)* are critical for the timing and integrity of myotube fusion and need to be expressed early in myoblast differentiation but shut down later or they inhibit myogenesis (51–55). All six genes were misregulated in at least five but none were affected in all patients (Fig. 4K). In general terms, these genes were upregulated in group 1, downregulated in group 3 and mixed in group 2. All six genes were upregulated in Bakay’s EDMD data, although only *NID1* and *HGF* were statistically significant at 5% FDR. Both were upregulated only in group 1 and downregulated in group 3. *PTN* showed a similar pattern of expression as *HGF* although the only statistically significant changes were for upregulation in group 1.

Several myogenic signaling pathways were altered such as MAPK, PI3K, BMP and Notch signaling, and several alternate differentiation pathways were de-repressed such as adipogenesis that could disrupt myotube formation and function (Fig. 2 and Supplementary Material, Table S3). Myogenesis and adipogenesis are two distinct differentiation routes from the same progenitor cells and whichever route is taken the other becomes repressed during normal differentiation (56,57). We previously showed that knockout of fat- or muscle-specific genome organizing NETs yield de-repression of the alternate differentiation pathway (17,58) and the Collas lab showed that Lamin A/C lipodystrophy point mutations yield de-repression of muscle differentiation genes in adipocytes (59). We now find here that adipogenesis genes are upregulated in both Bakay’s EDMD data and our data (Fig. 2). This is especially prominent for the five patients in group 1 while group 3 showing strong downregulation of a subset of the same genes and group 2 broadly looking like an intermediate of the other two groups (Fig. 4L and Supplementary Material, Figs S11 and S12), and thus could also contribute to the metabolic defect differences between patients.

### Splicing pathways uniformly altered in EDMD patients yield loss of muscle-specific splice variants

**Figure 5:**
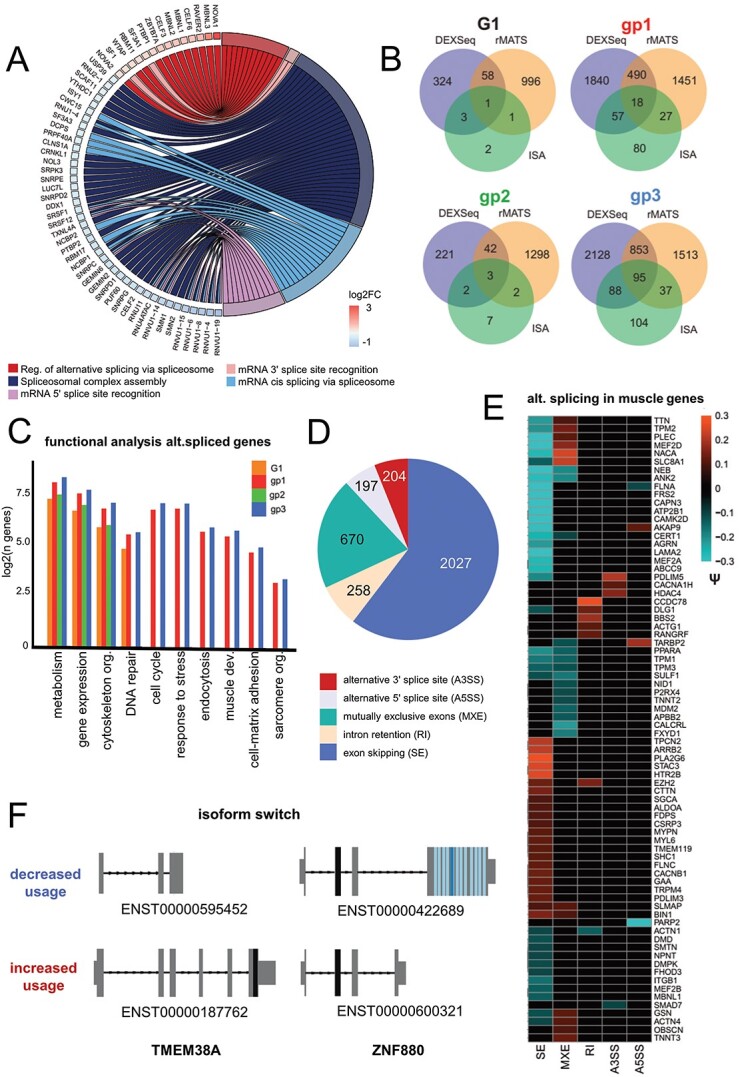
Analysis of splicing defects reveals loss of muscle-specific splice variants. (**A**) GOchord plot for misregulated splicing factors indicates the primary change is upregulation of alternative splicing and downregulation of constitutive splicing. Plot for gp1 is shown as a representative. DE genes log2FC > |0.15| and *P* < 0.05. (**B**) Venn diagrams of alternatively spliced genes predicted by rMATS, DEXSeq and ISA for all samples (G1) and the three subgroups (gp1, gp2 and gp3). Overlap between all three methods indicates genes with exon skipping events that lead to annotated isoform switches. (**C**) Bar chart for functional pathways reveals an enrichment in altered splice variants for pathways associated with metabolism, gene expression, cytoskeleton organization, DNA repair, proliferation/differentiation, stress, ECM/fibrosis/and sarcomere structure. Number of genes detected displayed as log2. (**E**) Pie chart of significant AS events with psi > |0.1| in gp1 detected with rMATS, which were used to scan for muscle specific-splice variants, shown as heatmap. AS events, alternative splicing events, SE = exon skipping, MXE = mutually exclusive exons, RI = intron retention, A3SS = alternative 3′ splice site, A5SS = alternative 5′ splice site. (**F**) Isoform switches as analyzed using isoformSwitchAnalyzer for ZNF880 and TMEM38A. ZNF880 shows the same isoform switch in all groups with preferential use of the shorter isoform that contains the KRAB domain (black), but not the zinc finger domain (blue). TMEM38A shows a clear switch from the muscle isoform to a shorter isoform.

Among the downregulated functional categories, mRNA splicing stood out with many genes uniformly downregulated in all patient samples (Fig. 2B and C and Supplementary Material, Fig. S14). Because of that we decided to investigate various subcategories and we found that there was a striking and uniform upregulation of factors supporting alternative splicing (AS) while constitutive splicing factors involved in spliceosome assembly and cis splicing are downregulated (Fig. 5A and Supplementary Material, Fig. S15). Expression changes of as little as 10% (log2FC > 0.1) have been shown to result in biologically relevant changes for vital proteins like kinases and splicing factors (60). We thus assume that upregulation and downregulation of whole spliceosome subcomplexes even in low log2FC ranges lead to significant splicing misregulation. Notably, snRNAs of the U1 spliceosomal subcomplex, responsible for 5’ SS recognition, constitute as much as 20% of all downregulated splicing factors (|log2FC| > 0.1, RNU1s and RNVU1s). Interestingly, a similar sharp cut-off between alternative and constitutive splicing has been reported in myotonic dystrophy (DM1/DM2) with similar genes being affected, namely *CELF*s, *MBNL*s, *NOVA*, *SMN1/2* and *SF3A1*, among others (61). DM1 is one of the best studied splicing diseases and shares typical muscular dystrophy symptomatology with EDMD, namely progressive muscle weakness and wasting, cardiac arrhythmia and contractures. Moreover, a number of splicing changes in DM1 and DM2 also occur in other muscular dystrophies (62). Of note, *MBNL3* is 4-fold transcriptionally upregulated in EDMD compared with controls. Its protein product impairs muscle cell differentiation in healthy muscle and thus needs to be downregulated upon differentiation onset (63).

Next, we performed splicing analysis to determine whether mis-splicing could drive some of the pathway alterations observed in the EDMD samples. For this purpose, we used three different methods: DEXSeq analyses exon usage, rMATS provides information about the five most common AS events and isoform Switch Analyzer (ISA) indicates which splicing events lead to annotated isoform switches. This revealed varying amounts of alternatively spliced genes in all samples and the three subgroups (Fig. 5B). Since every method focuses on a different event type/aspect of splicing, a higher amount of unique than overlapping genes is to be expected. Accordingly, an overlap of all three methods indicates genes with exon skipping events that lead to annotated isoform switches. The number of mis-spliced genes overlapping between the three algorithms was only 1 gene, *ZNF880*, in G1. In contrast, when analyzing group 1, 2 and 3 separately, each patient grouping had many mis-spliced genes identified by all three algorithms with 18 mis-spliced genes in the intersect for group 1, the group including half of the patients, and as much as 95 in group 3 (Supplementary Material, Table S4). These genes include Nesprin 3 (*SYNE3*), the splicing factor kinase *CLK1* and the chromatin regulator *HMGN3*, all of which are potentially contributing to EDMD, given their functions. All results can be found in Supplementary Material, Table S5. The rMATS analysis includes five AS events: exon skipping (SE), intron retention (RI), mutually exclusive exons (MXE), alternative 3′ splice site (A3SS) and alternative 5′ splice site (A5SS) usage. Using this comprehensive dataset, we searched for AS events that are significantly differentially used |(percent-spliced-in-(psi)-value| > 0.1 and *P*-value < 0.05, Supplementary Material, Table S5). Comparing AS event inclusion between control and EDMD samples, we find thrice as much intron retention in EDMD, while all other events are similarly included as excluded. We hypothesize that this could be a result of downregulated U1 snRNAs which are necessary for proper spliceosome assembly. Supporting the likely importance of the splicing pathway to the EDMD pathomechanism, pathway analysis on these genes revealed a strong enrichment for pathways associated with metabolism, gene expression and the cytoskeleton (Fig. 5C). Moreover, group 1 and group 3 display an enrichment for myogenesis and muscle contraction. Using a custom-made set of genes either specific or relevant for muscle development and structure (see Materials and Methods section), we then scanned all significant and differential AS events (Fig. 5D and Supplementary Material, Fig. S16). Notably, mis-splicing led to the absence of many muscle-specific splice variants (Fig. 5E), among them vital muscle structural genes like *TTN*, *TNNT3*, *NEB*, *ACTA4* and *OBSCN* as well as developmental regulators of the MEF2 family. Importantly, many of these genes mis-spliced in the EDMD patients are linked to a variety of other muscular dystrophies. For example, *TTN*, *CAPN3*, *PLEC* and *SGCA* are linked to Limb-Girdle muscular dystrophy (64–69), *DMD* is linked to Duchenne muscular dystrophy and Becker muscular dystrophy (70,71), and *BIN1*, *TNNT2/3* and *MBNL1* are mis-spliced in myotonic dystrophy (72) and all of these are mis-spliced and/or have missing muscle-specific splice variants in many of the patients in our cohort.

Intriguingly, one of the mis-spliced genes that also displays an isoform switch in group 3 is *TMEM38A* that has been linked to EDMD (10). The altered splicing map for *TMEM38A* reveals that not only is its expression highly elevated in EDMD patients (log2FC = 2.9) but also that the protein-coding isoform displays a higher usage relative to abundance compared with the non-coding isoform (Fig. 5F) Many other notable mis-spliced genes are involved in myotube fusion such as the previously mentioned *NID1* that is under spatial genome positioning control of NET39, another of the genome organizing NETs causative of EDMD. Most compellingly, three mis-spliced genes having to do with myogenesis/myotube fusion had muscle-specific splice variants absent in all patients (G1 rMATS). These were *CLCC1* whose loss yields muscle myotonia (73), *HLA-A/B* that disappears during myogenesis and is linked as a risk factor for idiopathic inflammatory myopathies (74,75), and *SMAD2* that shuts down myoblast fusion (76). The above examples were found in all patients within a particular group, but not always amongst all patients from the study or determined by all algorithms; however, there were also some mis-spliced genes that are potentially even more interesting because they were mis-spliced in all patients and with all three algorithms yielding the same results. One of these was *ZNF880*. While overall transcript numbers remained similar, the isoform predominantly expressed in control cells, ENST00000422689, is strongly downregulated in group 1 while the shorter isoform, ENST00000600321, is strongly upregulated (Fig. 5F). Interestingly, the dominant isoform in EDMD loses the zinc finger domain (light and dark blue) and is left with the repressive KRAB domain (black). Little is known about ZNF880 except that it has an unclear role in breast and rectal cancer (77,78), and additional experiments are necessary to elucidate its role in EDMD.

### miRNA-Seq analysis of EDMD patient cells

**Figure 6:**
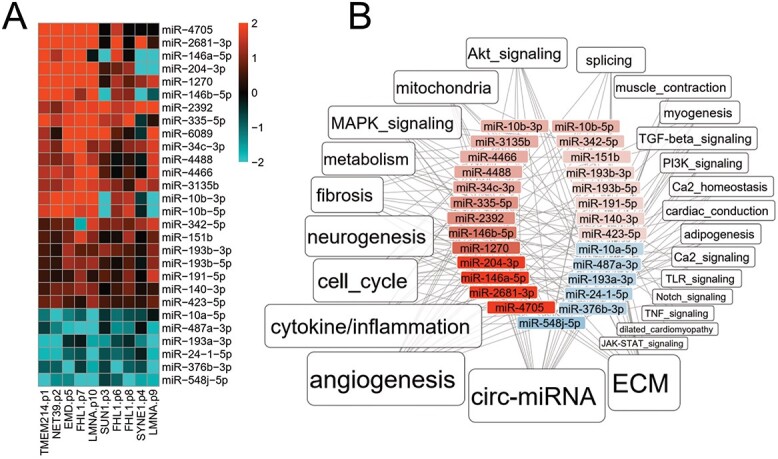
miRNA analysis. (**A**) Heatmap of miRNAs that had altered levels in EDMD patient cells compared with controls. Red indicates upregulated and blue downregulated with intensity according to the log2FC values. (**B**) Overview of functional categories linked to differentially expressed miRNAs in EDMD. The label size is proportional to the number of DE miRNAs associated with each category.

Changes in miRNA levels have been observed in a number of muscular dystrophies and are often used as biomarkers (79–81), but a comprehensive investigation of miRNA levels in EDMD has thus far not been engaged. Thus, the *in vitro* differentiated EDMD patient cells used for the preceding analysis were also analyzed by miRNA-Seq. We identified 28 differentially expressed miRNAs with some variation among patients (Fig. 6A). We extracted their putative targets from the miRDB database (http://mirdb.org) and selected those targets whose expression changed in the opposite direction of the miRNAs. Pathway analysis revealed misregulation of miRNAs largely associated with the same pathways that were misregulated from the RNA-Seq data, *e.g.* metabolism, ECM/fibrosis and signaling/differentiation (Fig. 6B and Supplementary Material, Table S6). More specifically, for metabolism 9 of the misregulated miRNA were linked to metabolic functions and with only partial overlap another 9 linked to mitochondria function, for ECM/fibrosis 19 of the misregulated miRNAs were linked to ECM and again with only partial overlap 10 to fibrosis and 13 to cytokines and inflammation. As noted before the ECM category in addition to potentially contributing to fibrosis is also relevant for myotube fusion along with cell cycle regulation that was targeted by 13 misregulated miRNAs and myogenesis that was targeted by 5 miRNAs. Several misregulated miRNAs had functions relating to alternative differentiation pathways with 4 relating to adipogenesis, 12 to neurogenesis, 17 to angiogenesis and for signaling there were 9 misregulated miRNAs affecting MAPK pathways, 8 for Akt signaling, 1 for JAK–STAT signaling, 5 for TGF-beta signaling, 2 for Notch signaling and 3 for TLR signaling. Interestingly, some misregulated miRNAs were also reported as being linked to disease states such as miR-140-3p to dilated cardiomyopathy through its repressive effect on the integrin metalloproteinase gene *ADAM17* (82). As well as working within cells, miRNAs are often detected within a circulating exosomal microvesicle population that can be harvested from blood serum. This makes them especially attractive as potential biomarkers when compared with more invasive biopsies, but a much larger sample size together with more clinical information will be required to clarify these as biomarkers.

### EDMD gene expression signature suggests relationships to other muscular dystrophies

The earlier Bakay study analyzed patient samples from other muscular dystrophies for comparison to EDMD. Several of the disorders show a high degree of pairwise similarity from a transcriptome point of view (Supplementary Material, Fig. S12A). In fact, many of the functional categories misregulated in EDMD are also altered in the same direction in several other muscular dystrophies, although with some differences (Supplementary Material, Fig. S12B). For example, collagens as a general category showed the highest correlations between EDMD and most other muscular disorders with the exception of hereditary spastic paraplegia, suggesting that the ECM is a major player in most muscular dystrophies. In contrast functional categories related to metabolism and mitochondrial function such as glycolysis, oxidative phosphorylation and mitophagy were more uniquely changed, i.e. weaker correlated between EDMD and other muscular disorders. Splicing also showed a weaker correlation with other muscular disorders compared with other muscle-specific functions (Supplementary Material, Fig. S12B). Because of the overall degree of similarity among neuromuscular diseases, and the fact that Bakay *et al.* used terminally differentiated muscle while our data come from a very early stage of differentiation *in vitro*, we reasoned that GSEA could be a sensitive method to compare the datasets. To this effect, we reanalyzed Bakay’s data and extracted the DE genes for each of the neuromuscular diseases. We then checked our data for enrichment of those specific genesets. We then plotted the GSEA normalized enrichment score against the –log10(*P*-value). This way we can visualize positive or negative associations on the *x*-axis, and the higher on the *y*-axis the higher the confidence (lower *P*-values).

**Figure 7:**
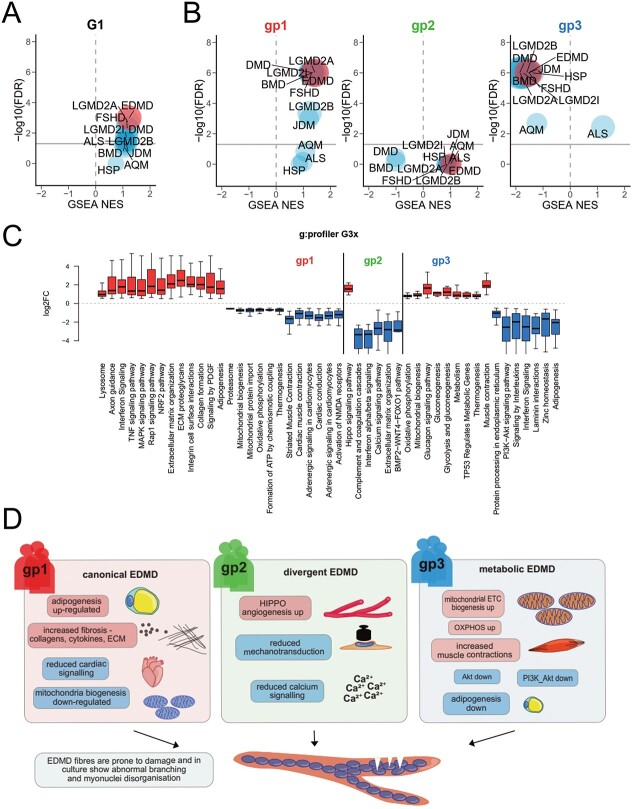
EDMD is distinct from other MDs. (**A**) Scatterplots from GSEA analysis comparing Bakay data for several muscular dystrophies to the new patient data from this study (all 10 patients as a single group = G1). On the *x*-axis, the normalized enriched score (NES) is a measurement of the enrichment of the DE geneset identified in our study compared with each of the diseases in the Bakay study. The *y*-axis shows the –log10(FDR), which is a measurement of statistical confidence. The gray horizontal line marks the 5% FDR threshold. Muscular dystrophies from the Bakay study are EDMD, Limb-Girdle muscular dystrophy 2A (LGMD2A), Limb-Girdle muscular dystrophy 2B (LGMD2B), Limb-Girdle muscular dystrophy 2I (LGMD2I), fascioscapulohumeral muscular dystrophy (FSHD), Duchenne muscular dystrophy (DMD), Becker muscular dystrophy (BMD), juvenile dermatomyositis (JDM), acute quadriplegic myopathy (AQM), amyotrophic lateral sclerosis (ALS) and hereditary spastic paraplegia (HSP). (**B**) Same as (A), for each individual patient subgroup. (**C**) Box plot of log2FC values for differentially expressed genes within significantly enriched functional categories for each patient subgroup compared with the rest, using g:Profiler. (**D**) Each patient subgroup was relatively more enriched for certain functional pathways than other subgroups, suggesting that treatments for example targeting different metabolic pathways for groups 1 and 3 might partially ameliorate some patient difficulties.

When we looked at all patients as a single group (G1), EDMD was the best match, with the highest score and lowest *P*-value (FDR 0.001), although unsurprisingly a few other diseases came very close, notably LGMD2A and facioscapulohumeral muscular dystrophy (FSHD) (Fig. 7A). This indicates that despite the differences in the individual DE genes between the mature muscle data from Bakay’s EDMD geneset and our early *in vitro* differentiation geneset, a clear EDMD gene expression signature was displayed in our data. The next best match is Limb-Girdle muscular dystrophy 2A (LGMD2A), which shares some symptomatology with EDMD including muscle wasting, contractures and mild cardiac conduction defects although cardiac involvement is infrequent (83). The differences in gene signatures that broke down the 10 patients into three EDMD patient subgroups could reflect an underlying cause of clinical disease spectrum or indicate that a group may not be adequately classified as EDMD. Therefore, we performed the same GSEA analysis on each subgroup separately. Group 1, which had both classic emerin and lamin A EDMD mutations, showed an even better match with the Bakay EDMD group which was again very close to LGMD2A but also to DMD, Becker muscular dystrophy (BMD), FSHD and Limb-Girdle muscular dystrophy 2I (LGMD2I) (Fig. 7B). LGMD2B was still separate and closer to juvenile dermatomyositis (JDM). For group 2, none of the diseases matched at 5% FDR, although the Bakay EDMD set remained the most like our set. Interestingly, two diseases exhibited an anti-correlation: DMD and BMD, which are both caused by mutations in the dystrophin gene *DMD*. In contrast, group 3 appeared to be the most distinct and in many ways opposite to group 1, which is a pattern that was often observed in the functional gene subsets analyzed (Supplementary Material, Figs S5–S11). Group 3 was anti-correlated with EDMD and most of the other muscular dystrophies, while the neurogenic amyotrophic lateral sclerosis appeared as the best match, possibly suggesting a neuronal bias in this group (Fig. 7B). This is further supported by the appearance of axonal neuropathy, ataxia, undergrowth and speech problems in one of the two patients from this group (patient 4, *SYNE1*; Table 1), while none of the others exhibited any signs of neuropathy.

The relationship of the patient groups segregated by gene signatures to potential differences in clinical presentation is underscored by the functional pathways enriched in each group over the others (Fig. 7C). Group 1 showed a strong enrichment of pathways associated with ECM and fibrosis, such as interferon signaling, TNF signaling, ECM organization, ECM proteoglycans, integrin cell surface interactions, collagen formation and signaling by PDGF all upregulated. Adipogenesis was also particularly promoted in group 1 compared with the others, and cardiac conduction defects were also highlighted. Group 2 was more uniquely associated with Hippo signaling and BMP2-WNT4-FOXO1 pathway and had fewer links to ECM and fibrosis. Group 3 was more uniquely associated with metabolism, particularly upregulation of oxidative phosphorylation, mitochondrial biogenesis, glucagon signaling pathway, gluconeogenesis, glycolysis and gluconeogenesis, metabolism, TP53 regulates metabolic genes and thermogenesis pathways. This would suggest that group 1 pathophysiology may have more characteristics of fibrosis and altered myofibers, while group 2 may have more differentiation or mechanosignaling defects and group 3 more metabolic defects (Fig. 7D).

## Discussion

Attempts to identify the EDMD pathomechanism or clinical biomarkers purely through gene expression signatures are limited because there is too little uniformity in differential gene expression between all patients, although there have been some promising reports using miRNA profiling or detection of cytokines in serum (81,84). We therefore engaged a functional pathway analysis using *in vitro* differentiated myotubes derived from 10 unrelated EDMD patients with known mutations in seven EDMD-linked genes. While it is difficult to detect many individual genes that were uniformly changed in all patients, we found many pathways that were affected in all patients. Thus, although different genes may have been targeted in different patients, the same functional pathway would be disrupted and thus yield a pathology with similar clinical features. Many pathways were disrupted when we re-analyzed data from the previously published Bakay study (20) and we postulated that, as they just analyzed mutations in two of the over two dozen genes linked to EDMD, analyzing a larger set of linked genes might narrow down the number of pathways to highlight those most relevant to EDMD pathophysiology. Indeed, when we considered a wider set of patients with mutations in seven different genes the set of affected pathways narrowed to the point that we could identify four likely candidate umbrella pathways.

These four umbrella pathways all make sense for contributing to or even driving the EDMD pathomechanism (85). Disruption of metabolism pathways was consistent with the significantly reduced glycolysis and mitochondrial respiration output we showed in patient myoblasts compared with controls and it makes sense that this could lead to fatigue, weakness and muscle atrophy. ECM changes and fibrosis pathways are consistent with pathology observed in EDMD and similarly could drive some of the initial pathology and, as fibrosis accumulates, contribute to disease progression. De-repression of genes from alternate differentiation pathways and defects in myogenesis through disrupted signaling pathways and cell cycle regulation could generate aberrant myotubes to yield pathology. Finally, the last disrupted pathway of splicing yields a loss of muscle-specific splice variants that could impact on all three preceding pathways.

There is much scope for intersection between the four highlighted pathways altered in all sampled EDMD patient cells. For example, amongst the de-repressed differentiation pathways was adipogenesis that could also have impact on the metabolism pathway. Even amongst the few genes that were uniformly altered in all patients sampled, though not originally obvious, a more detailed reading of the literature leads to intersections with these pathways. For example, while the *MYH14* general upregulation did not make obvious sense for muscle defects since it is not part of the contractile machinery, it has been shown that a mutation in *MYH14* disrupts mitochondrial fission in peripheral neuropathy (31). Thus, *MYH14* could potentially feed into the mitochondrial deficits noted in the patient cells. Many of the miRNAs found to be altered in the patients feed into several of these pathways. For example, miR-2392 that is increased in all patients downregulates oxidative phosphorylation in mitochondria (86) but at the same time also is reported to promote inflammation (87). miR-140 that is up in all groups has roles in fibrosis through collagen regulation (88), is pro-adipogenic (89) and inhibits skeletal muscle glycolysis (90). miRNAs could also be used potentially prognostically between the different groups as for example miR-146a is upregulated in group 1, unchanged in group 2 and downregulated in group 3. This miRNA has a strong effect on inflammation and has been implicated in fibrosis in the heart (91). miRNAs show some promise as biomarkers, especially if they could be isolated from circulating exosome vesicles in serum. Several miRNAs have been proposed as markers for lamin A/C-associated muscular dystrophies, targeting functions such as muscle repair through TGF-beta and Wnt signaling. Some of those were also identified in our study, such as miR-335, which plays a role in muscle differentiation (81). Other miRNAs identified in that study appear to be misregulated only in one subgroup of patients but not others. For example, miR-100 and miR-127-3p are misregulated in group 1 only, while miR-136, miR-376c and miR-502-3p are only misregulated in group 3, and miR-148a is upregulated in group 1 but downregulated in group 3. Because there is so much functional overlap between miRNA targets and the pathways noted from the RNA-Seq analysis, it is unclear to what extent the gene expression changes observed could be indirect from the misregulated miRNAs. Nonetheless, there are four core functions targeted by multiple mechanisms that we argue are likely to be central to the core EDMD pathomechanism. Interestingly, the literature is filled with many examples of mutation or loss of different splicing factors causing muscle defects though no individual mis-spliced gene was identified as mediating these effects. Similarly, in myotonic dystrophy type 1 (DM1) there are many mis-spliced genes thought to contribute to the disease pathology (72). For example, the splicing factor SRSF1 that is down in most patients is important for neuromuscular junction formation in mice (92). It has to be noted that while the present study used *in-vitro* differentiated myotubes, these pathways may cross-talk not just at the level of gene expression in the myotubes themselves, but also through effects determined *in vivo* by the muscle microenvironment which may vary depending on the activation of inflammatory pathways, for example.

How so many genes become misregulated has not been experimentally proven, but for lamin A/C, emerin, Sun1, nesprin, TMEM214 and PLPP7/NET39, the fact that mutations to all individually yield many hundreds of gene expression changes with considerable overlap strongly suggests that they function in a complex at the nuclear envelope to direct genome organization. Knockdown of Tmem214 and NET39 as well as several other muscle-specific NETs each alters the position and expression of hundreds of genes (17). Separately it was found that lamins and NETs, including emerin, function together in distinct complexes involved in tethering genes to the nuclear envelope in fibroblasts (93) and in muscle cells (94). Thus, disruption of emerin, lamin A/C or any other component of these tethering complexes could yield sufficiently similar gene/pathway expression changes to yield the core clinical features of EDMD. We propose that the different muscle-specific NETs give specificity to a complex containing lamin A/C and emerin and that Sun1 and nesprin proteins can indirectly impact on these complexes through mediating mechanosignal transduction and FHL1 in interpreting such signals. Since 15% of all genes changing here were affected by at least one of the muscle-specific genome-organizing NETs that were tested by knockdown, this would provide a core set of genome organization and expression changes to cause the core EDMD pathology. Since the majority of genes affected by each NET tested were unique to that NET with the exception of Tmem214, this could account for other gene expression changes that drive the segregation into subgroups which could in turn contribute to clinical variation. This interpretation is consistent with the numbers of genes changing for mutations in different nuclear envelope proteins (Fig. 1B). That the genome organizing NET mutations yielded fewer genes changing than the lamin A/C mutations may be because lamin A/C mutations disrupt multiple genome tethering complexes and thus affect more genes. The segregation of the two *LMNA* mutation gene signatures into separate groups might reflect separate complexes for lamin C or each mutation disrupting different sets of complexes. In either case, the extreme differences in lamin mutations gene expression profiles is not entirely surprising as different lamin mutations also exhibited large differences in studies of nuclear mechanics (95); so this could also impact on mechanosignal transduction. The Sun1 mutation may have affected fewer genes because of redundancy with Sun2 in its mechanosignal transduction function while the Nesprin 1 mutation may have had more genes changing because it is more central to mechanosignal transduction. More work is needed to clarify on all these possibilities.

The FHL1 mutations add another level of complexity to EDMD as there are several splice variants of FHL1 and only the B variant (ENST00000394155) targets to the nuclear envelope (96). That EDMD is a nuclear envelope disorder is underscored by the fact that none of the FHL1 mutations occur in exons found in the much shorter C variant (ENST00000618438) and the patient 8 mutation p.V280M is in an exon unique to FHL1B. Thus, the nuclear envelope splice variant is the only one that could yield pathology in all patients, though some of the variation could come from one of the patients also expressing the mutant A splice variant (ENST00000543669).

While further work is needed to validate the correlations between the gene expression profile subgroupings and their clinical presentation and disease progression, our finding of such distinct gene expression profiles amongst clinically diagnosed EDMD patients argues that the currently used clinical phenotype spectrum umbrella of the EDMD classification may be too broad and it might be reclassified in more precise subtypes. What is clear is that the original classifications of EDMD subtypes based just on the mutated gene often allow for cases with very dissimilar gene profiles to be classified together, while similar gene signature cases are classified as separate classes. The two mutations in *LMNA* yielded changes in gene expression profiles that were far more different from one another than the group 1 lamin A mutation gene profile was from the TMEM214, NET39, emerin and FHL1 mutation gene signatures. Similarly, the FHL1 p.C224W mutation yielded greater gene expression differences from the other two FHL1 mutations than it did for the other proteins in group 1. Thus, EDMD might be better classified by similarities in gene expression profiles than by the particular gene mutated and our study shows proof of principle for this, even if the groupings may change slightly once a larger patient cohort can be established and examined. Regardless, these groups have distinctive gene expression and miRNA signatures that could be used as biomarkers both diagnostically and perhaps prognostically. To get to that point will require a more comprehensive modern description of clinical Gestalt phenotypes including *e.g.* imaging datasets and disease progression timelines deciphering unique groups. Importantly, and regardless of the disease nomenclature, the different pathways we found enriched for in each subgroup could be converted to clinical recommendations based on the much more conserved individual gene expression changes for each subgroup (Fig. 7C). For example, EDMD patients have been considered by some clinicians to be at risk for malignant hyperthermia (97), though a consensus was never achieved. Our data show that the three genes currently associated with malignant hyperthermia (*RYR1*, *CACNA1S* and *STAC3*) are all misregulated in groups 1 and 3, but not group 2 (Supplementary Material, Fig. S17). Thus, checking expression of these genes might indicate whether a patient is likely to be at risk or not. Finally, an additional new aspect coming up from our datasets is that it might be worth further investigating the role of splicing in muscle differentiation because it might be of wider relevance to muscular dystrophy beyond DM and EDMD.

## Material and Methods

### Patient materials

The sources of patient samples were the Muscle Tissue Culture Collection (MTCC) at the Friedrich-Baur-Institute (Department of Neurology, Ludwig-Maximilians-University, Munich, Germany) and the MRC Centre for Neuromuscular Disorders Biobank (CNDB) in London.

### Ethical approval and consent to participate

All materials were obtained with written informed consent of the donor at the CNDB or the MTCC. Ethical approval of the rare diseases biological samples biobank for research to facilitate pharmacological, gene and cell therapy trials in neuromuscular disorders is covered by REC reference 06/Q0406/33 with MTA reference CNMDBBL63 CT-2925/CT-1402, and for this particular study was obtained from the West of Scotland Research Ethics Service (WoSRES) with REC reference 15/WS/0069 and IRAS project ID 177946. The study conduct and design complied with the criteria set by the Declaration of Helsinki.

### Myoblast culture and in vitro differentiation into myotubes

Myoblasts were grown in culture at 37°C and 5% CO_2_ using a ready to use formulation for skeletal muscle (PELOBiotech #PB-MH-272-0090) and maintained in subconfluent conditions. In order to induce differentiation, the cells were grown to confluency and 24 h later the growth medium replaced with skeletal muscle differentiation medium (Cell Applications #151D-250). The differentiation medium was replaced every other day. Myotubes were selectively harvested after 6 days by partial trypsinization followed by gentle centrifugation (Supplementary Material, Fig. S1). Each differentiation experiment was performed in triplicate. To avoid batch effects, experiments were distributed over several weeks, so that no two samples or replicates were differentiated at the same time. Myotubes were stored in Trizol at –80°C until all samples were collected.

### RNA extraction

Myotubes were stored in Trizol at –80°C until all samples were available. Subsequent steps were performed simultaneously for all samples. Total RNA was extracted from each sample and separated into a high molecular weight fraction (>200 nt, for mRNA-Seq) and a low molecular weight fraction (<200 nt, for miRNA-Seq) with the Qiagen RNeasy (#74134) and miRNeasy (#1038703) kits, according to the manufacturer’s instructions. RNA quality was assessed with a Bioanalyzer (Agilent Technologies), and all samples had an RIN > 7, with an average of 9.4 (Supplementary Material, Table S1).

### mRNA-Seq analysis

Between 3 and 5 μg of total RNA were sent to Admera Health LLC (NJ, USA) for sequencing in paired-end mode, 2x 150 nucleotides, using an Illumina HiSeq 2500 sequencer. The sequencing library was prepared with the NEBNext Ultra II kit, with RiboZero rRNA depletion (NEB #E7103, Illumina #20040526). Between 60 and 90 million paired end reads were obtained from each sample and mapped to the human genome (Hg38) with STAR v2.7.5a (98) using default parameters. Mapping quality was assessed with FastQC v0.11.9 (https://www.bioinformatics.babraham.ac.uk/projects/fastqc). Sequencing adaptors were removed with trimmomatic v0.35 (99). Low-quality reads and mitochondrial contaminants were removed, leaving on average 70 million useful reads per sample (Supplementary Material, Table S1). Differential expression analysis was performed in R with DESeq2 v1.32.0 (100) after transcript quantitation with Salmon v1.4.0 (101). We used an FDR threshold of 5% for differential expression.

### miRNA-Seq analysis

miRNA was sent to RealSeq Biosciences Inc. (SC, USA) for sequencing using an Illumina NextSeq 500 v2 sequencer in single end mode, 1 × 75 nucleotides. The sequencing library was prepared with Somagenics’ Low-bias RealSeq-AC miRNA library kit (#500–00012) and quality assessed by Tapestation (Lab901/Agilent). On average, 5 million good quality reads were obtained per sample. Mapping and quality trimming was performed using the NextFlow nf-core/smrnaseq pipeline (https://nf-co.re/smrnaseq) with default parameters, which summarizes the reads per miRNA using the annotations from mirTop (https://github.com/miRTop/mirtop). Differential expression analysis was performed in R with DESeq2 v1.32.0. We used an FDR threshold of 0.2 for differential expression. Putative miRNA targets were extracted from miRDB (https://mirdb.org/) for each differentially expressed miRNA and their expression compared against the miRNA. We kept as potential targets those genes whose expression changed in the opposite direction of the miRNA.

### Bakay muscular dystrophy dataset analysis

Normalized (MAS5.0) microarray transcriptome data for a panel of 11 muscular dystrophies and healthy controls were downloaded from the Gene Expression Omnibus database (GEO), accession GSE3307 (https://www.ncbi.nlm.nih.gov/geo/). Differential expression analysis comparing each disease to the controls was performed using Limma 3.48.1 (102). We used an FDR threshold of 5% for differential expression.

### Functional analyses

Functional analyses were performed with g:Profiler (103) and Gene Set Enrichment Analysis (GSEA v4.1.0) (35) tools. g:Profiler was used to determine enriched categories within a set of DE genes, with an FDR of 5% as threshold. GSEA was performed with default parameters, in particular using ‘Signal2Noise’ as ranking metric and ‘meandiv’ normalization mode. Redundancy in category lists was reduced by comparing the similarity between each pair of enriched categories using Jaccard similarity coefficients. Hierarchical clustering (k-means) was then applied to the resulting matrix in order to identify groups of similar functional categories, and a representative from each group chosen. Full unfiltered results are shown in Supplementary Material, Table S3. Tissue-specific gene enrichment analysis was evaluated with TissueEnrich (104).

For the miRNA-Seq experiments, functional analysis was first performed using g:profiler on the set of DE miRNA genes. Then, putative targets for each miRNA were extracted and their expression compared with the relevant miRNA. Putative targets whose expression was not altered in the opposite direction as the miRNA were removed from the list. Significant functions were displayed using Cytoscape v3.8.2 (105), with the size of the functional labels proportional to the number of miRNAs assigned to each function.

### Real-time metabolic measurements

Metabolic measurements on primary human myoblast cultures were performed using a Seahorse XFp Extracellular Flux Analyzer (Agilent Technologies). For this, myoblasts of matched passage number were seeded in XFp Cell Culture Miniplates (103025-100, Agilent Technologies) at a density of 1.5 × 10^4^ cells per well. Cell density was assessed using an automated cell counter (TC20, BioRad). Oxygen consumption rates and extracellular acidification rates were measured using the Mito Stress Test Kit and the Glycolysis Stress Test Kit (Agilent Technologies #103020-100), respectively, according to the manufacturer’s instructions. Samples were measured in triplicates and each measurement was repeated between two and four times. Data were normalized to the number of cells and analyzed for each well.

### Fuel dependency tests

Glucose dependency and fatty acid dependency were determined according the instruction of Agilent Seahorse XF Mito Fuel Flex Test kit (Agilent Technologies #103260-100). The glutamine dependency was determined from the glucose and fatty acid measurements.

### Mitochondrial gene quantification

Reverse transcription of RNA was performed using the QuantiTecT Reverse Transcription Kit (Qiagen #205311) following the manufacturer’s instructions. For the reaction we used the SYBR® Green Master Mix (Bio-Rad #1725150) and samples were run and measured on CFX Connect™ (Bio-Rad). As genome reference gene *B2M* (FP: 5‘-TGCTGTCTCCATGTTTGATGTATCT-3′; RP: 5‘-TCTCTGCTCCCCACCTCTAAGT-3′) (106). Primer sequences for the mitochondrial genome were: FP: 5′-TTAACTCCACCATTAGCACC-3′; RP: 5′-GAGGATGGTGGTCAAGGGA-3′ (107). Samples were analyzed using the delta Ct method.

### Splice site prediction analysis

Raw data was mapped to the human genome assembly GRCh38 (hg38) and sorted by coordinate using STAR 2.7.9a (98) for analysis in DESeq2 (100) and DEXSeq (108), trimmed using an in-built trimming function for rMATS (109) or counted using Kallisto 0.48.0 (110) for isoformSwitchAnalyzer (ISA) (111). All analyses were performed for G1, gp1, gp2 and gp3 separately. Visualizations were conducted in R version 4.1.2.

DESeq2: Mapped reads were counted using FeatureCounts, then analyzed using DESeq2 (100). The R package fgsea was used for GSEA, and genes were assigned to biological pathways retrieved from MSigDB v7.5.1 (c5.go.bp.v7.5.1.symbols.gmt, 7658 gene sets, (35)). Splicing pathways and their genes were plotted using GOPlot (112).

DEXseq: Mapped reads were counted using the in-built DEXseq counting function in Python 3.9. Standard DEXseq workflow was followed. Exons with |logFCs| > 1 and *P*-values < 0.05 were set to be significantly different.

rMATS: Standard workflow was followed, and code was executed in Python 2.7. Results were analyzed in R and set to be significantly differentially spliced with |psi-values| > 0.1 and *P*-values < 0.05. Pie charts displaying the distribution of event usage were generated for all groups (Supps. something). GO term enrichment analysis was performed using g:profiler2 (113). All events were searched for muscle-specific genes using a set of 867 genes relevant for muscle system process, development, structure and contraction, combined from GO terms (GO:0003012, GO:0006936, GO:0055001 and GO:0061061).

ISA: Kallisto counts were read into R and standard ISA procedure was followed, including splicing analysis using DEXseq, coding potential using CPC 2.0 (114), domain annotation using HmmerWeb Pfam 35.0 (115), signal peptides using SignalP 5.0 (116) and prediction of intrinsically unstructured proteins using IUPred2A (117). In-built visualization tools were used for splicing maps.

## Data availability

Bakay *et al.* muscular dystrophy dataset is available at NCBI GEO with accession GSE3307.

RNA-Seq and miRNA-Seq datasets have been deposited at NCBI GEO with accession GSE204804 and GSE204826, respectively.

*Conflict of Interest statement*: The authors have no conflicts of interest to declare.

## Supplementary Material

Supplemental_Figures_and_legends_ddac264

Supplemental_Table_S1_ddac264

Supplemental_Table_S2_ddac264

Supplemental_Table_S3_ddac264

Supplemental_Table_S4_ddac264

Supplemental_Table_S5_ddac264

Supplemental_Table_S6_ddac264

## References

[1] Norwood, F.L.M., Harling, C., Chinnery, P.F., Eagle, M., Bushby, K. and Straub, V. (2009) Prevalence of genetic muscle disease in Northern England: in-depth analysis of a muscle clinic population. Brain, 132, 3175–3186. 19767415 10.1093/brain/awp236 PMC4038491

[2] Bonne, G., Mercuri, E., Muchir, A., Urtizberea, A., Becane, H.M., Recan, D., Merlini, L., Wehnert, M., Boor, R., Reuner, U. et al. (2000) Clinical and molecular genetic spectrum of autosomal dominant Emery-Dreifuss muscular dystrophy due to mutations of the lamin A/C gene. Ann. Neurol., 48, 170–180. 10939567

[3] Mercuri, E., Poppe, M., Quinlivan, R., Messina, S., Kinali, M., Demay, L., Bourke, J., Richard, P., Sewry, C., Pike, M. et al. (2004) Extreme variability of phenotype in patients with an identical missense mutation in the lamin A/C gene: from congenital onset with severe phenotype to milder classic Emery-Dreifuss variant. Arch. Neurol., 61, 690–694. 15148145 10.1001/archneur.61.5.690

[4] Rankin, J., Auer-Grumbach, M., Bagg, W., Colclough, K., Nguyen, T.D., Fenton-May, J., Hattersley, A., Hudson, J., Jardine, P., Josifova, D. et al. (2008) Extreme phenotypic diversity and nonpenetrance in families with the LMNA gene mutation R644C. Am. J. Med. Genet. A, 146A, 1530–1542. 18478590 10.1002/ajmg.a.32331

[5] Hoeltzenbein, M., Karow, T., Zeller, J.A., Warzok, R., Wulff, K., Zschiesche, M., Herrmann, F.H., Grosse-Heitmeyer, W. and Wehnert, M.S. (1999) Severe clinical expression in X-linked Emery-Dreifuss muscular dystrophy. Neuromuscul. Disord., 9, 166–170. 10382910 10.1016/s0960-8966(98)00120-5

[6] Emery, A.E. and Dreifuss, F.E. (1966) Unusual type of benign x-linked muscular dystrophy. J. Neurol. Neurosurg. Psychiatry, 29, 338–342. 5969090 10.1136/jnnp.29.4.338 PMC1064196

[7] Emery, A.E. (1989) Emery-Dreifuss syndrome. J. Med. Genet., 26, 637–641. 2685312 10.1136/jmg.26.10.637 PMC1015715

[8] Knoblauch, H., Geier, C., Adams, S., Budde, B., Rudolph, A., Zacharias, U., Schulz-Menger, J., Spuler, A., Yaou, R.B., Nurnberg, P. et al. (2010) Contractures and hypertrophic cardiomyopathy in a novel FHL1 mutation. Ann. Neurol., 67, 136–140. 20186852 10.1002/ana.21839

[9] Bonne, G. and Quijano-Roy, S. (2013) Emery-Dreifuss muscular dystrophy, laminopathies, and other nuclear envelopathies. Handb. Clin. Neurol., 113, 1367–1376. 23622360 10.1016/B978-0-444-59565-2.00007-1

[10] Meinke, P., Kerr, A.R.W., Czapiewski, R., de Las Heras, J.I., Dixon, C.R., Harris, E., Kolbel, H., Muntoni, F., Schara, U., Straub, V. et al. (2020) A multistage sequencing strategy pinpoints novel candidate alleles for Emery-Dreifuss muscular dystrophy and supports gene misregulation as its pathomechanism. EBioMedicine, 51, 102587. https://doi.org/10.1016. 31862442 10.1016/j.ebiom.2019.11.048 PMC7000448

[11] Meinke, P. (2018) Molecular genetics of Emery–Dreifuss muscular dystrophy. eLS. 10.1002/9780470015902.a0022438.pub2.

[12] Bridger, J.M., Foeger, N., Kill, I.R. and Herrmann, H. (2007) The nuclear lamina. Both a structural framework and a platform for genome organization. FEBS J., 274, 1354–1361. 17489093 10.1111/j.1742-4658.2007.05694.x

[13] Worman, H.J. and Bonne, G. (2007) "Laminopathies": a wide spectrum of human diseases. Exp. Cell Res., 313, 2121–2133. 17467691 10.1016/j.yexcr.2007.03.028 PMC2964355

[14] Meinke, P., Mattioli, E., Haque, F., Antoku, S., Columbaro, M., Straatman, K.R., Worman, H.J., Gundersen, G.G., Lattanzi, G., Wehnert, M. and Shackleton, S. (2014) Muscular dystrophy-associated SUN1 and SUN2 variants disrupt nuclear-cytoskeletal connections and myonuclear organization. PLoS Genet., 10, e1004605. 25210889 10.1371/journal.pgen.1004605 PMC4161305

[15] Zhang, Q., Bethmann, C., Worth, N.F., Davies, J.D., Wasner, C., Feuer, A., Ragnauth, C.D., Yi, Q., Mellad, J.A., Warren, D.T. et al. (2007) Nesprin-1 and -2 are involved in the pathogenesis of Emery-Dreifuss muscular dystrophy and are critical for nuclear envelope integrity. Hum. Mol. Genet., 16, 2816–2833. 17761684 10.1093/hmg/ddm238

[16] Piccus, R. and Brayson, D. (2020) The nuclear envelope: LINCing tissue mechanics to genome regulation in cardiac and skeletal muscle. Biol. Lett., 16, 20200302. 32634376 10.1098/rsbl.2020.0302 PMC7423042

[17] Robson, M.I., de Las Heras, J.I., Czapiewski, R., Le Thanh, P., Booth, D.G., Kelly, D.A., Webb, S., Kerr, A.R. and Schirmer, E.C. (2016) Tissue-specific gene repositioning by muscle nuclear membrane proteins enhances repression of critical developmental genes during myogenesis. Mol. Cell, 62, 834–847. 27264872 10.1016/j.molcel.2016.04.035 PMC4914829

[18] Wilkie, G.S., Korfali, N., Swanson, S.K., Malik, P., Srsen, V., Batrakou, D.G., de las Heras, J., Zuleger, N., Kerr, A.R., Florens, L. and Schirmer, E.C. (2011) Several novel nuclear envelope transmembrane proteins identified in skeletal muscle have cytoskeletal associations. Mol. Cell. Proteomics, 10, M110.003129. 10.1074/mcp.M110.003129 PMC3016689 20876400

[19] Zuleger, N., Boyle, S., Kelly, D.A., de Las Heras, J.I., Lazou, V., Korfali, N., Batrakou, D.G., Randles, K.N., Morris, G.E., Harrison, D.J. et al. (2013) Specific nuclear envelope transmembrane proteins can promote the location of chromosomes to and from the nuclear periphery. Genome Biol., 14, R14. 23414781 10.1186/gb-2013-14-2-r14 PMC4053941

[20] Bakay, M., Wang, Z., Melcon, G., Schiltz, L., Xuan, J., Zhao, P., Sartorelli, V., Seo, J., Pegoraro, E., Angelini, C. et al. (2006) Nuclear envelope dystrophies show a transcriptional fingerprint suggesting disruption of Rb-MyoD pathways in muscle regeneration. Brain, 129, 996–1013. 16478798 10.1093/brain/awl023

[21] Melcon, G., Kozlov, S., Cutler, D.A., Sullivan, T., Hernandez, L., Zhao, P., Mitchell, S., Nader, G., Bakay, M., Rottman, J.N., Hoffman, E.P. and Stewart, C.L. (2006) Loss of emerin at the nuclear envelope disrupts the Rb1/E2F and MyoD pathways during muscle regeneration. Hum. Mol. Genet., 15, 637–651. 16403804 10.1093/hmg/ddi479

[22] Bione, S., Maestrini, E., Rivella, S., Mancini, M., Regis, S., Romeo, G. and Toniolo, D. (1994) Identification of a novel X-linked gene responsible for Emery-Dreifuss muscular dystrophy. Nat. Genet., 8, 323–327. 7894480 10.1038/ng1294-323

[23] Bonne, G., Di Barletta, M.R., Varnous, S., Becane, H.M., Hammouda, E.H., Merlini, L., Muntoni, F., Greenberg, C.R., Gary, F., Urtizberea, J.A. et al. (1999) Mutations in the gene encoding lamin A/C cause autosomal dominant Emery-Dreifuss muscular dystrophy. Nat. Genet., 21, 285–288. 10080180 10.1038/6799

[24] Gueneau, L., Bertrand, A.T., Jais, J.P., Salih, M.A., Stojkovic, T., Wehnert, M., Hoeltzenbein, M., Spuler, S., Saitoh, S., Verschueren, A. et al. (2009) Mutations of the FHL1 gene cause Emery-Dreifuss muscular dystrophy. Am. J. Hum. Genet., 85, 338–353. 19716112 10.1016/j.ajhg.2009.07.015 PMC2771595

[25] Ruiz, E., Penrose, H.M., Heller, S., Nakhoul, H., Baddoo, M., Flemington, E.F., Kandil, E. and Savkovic, S.D. (2020) Bacterial TLR4 and NOD2 signaling linked to reduced mitochondrial energy function in active inflammatory bowel disease. Gut Microbes, 11, 350–363. 31063017 10.1080/19490976.2019.1611152 PMC7524318

[26] Campbell, I.L., Bizilj, K., Colman, P.G., Tuck, B.E. and Harrison, L.C. (1986) Interferon-gamma induces the expression of HLA-A, B, C but not HLA-DR on human pancreatic beta-cells. J. Clin. Endocrinol. Metab., 62, 1101–1109. 3084532 10.1210/jcem-62-6-1101

[27] Browne, S.K., Roesser, J.R., Zhu, S.Z. and Ginder, G.D. (2006) Differential IFN-gamma stimulation of HLA-A gene expression through CRM-1-dependent nuclear RNA export. J. Immunol., 177, 8612–8619. 17142760 10.4049/jimmunol.177.12.8612

[28] Behrends, C., Sowa, M.E., Gygi, S.P. and Harper, J.W. (2010) Network organization of the human autophagy system. Nature, 466, 68–76. 20562859 10.1038/nature09204 PMC2901998

[29] Choi, B.-O., Kang, S.H., Hyun, Y.S., Kanwal, S., Park, S.W., Koo, H., Kim, S.-B., Choi, Y.-C., Yoo, J.H., Kim, J.-W. et al. (2011) A complex phenotype of peripheral neuropathy, myopathy, hoarseness, and hearing loss is linked to an autosomal dominant mutation in MYH14. Hum. Mutat., 32, 669–677. 21480433 10.1002/humu.21488 PMC3103632

[30] Iyadurai, S., Arnold, D.A., Kissel, J.T., Ruhno, C., McGovern, V.L., Snyder, P.J., Prior, T.W., Roggenbuck, J., Burghes, A.H. and Kolb, S.J. (2017) Variable phenotypic expression and onset in MYH14 distal hereditary motor neuropathy phenotype in a large, multigenerational North American family. Muscle Nerve, 56, 341–345. 27875632 10.1002/mus.25491 PMC5440221

[31] Almutawa, W., Smith, C., Sabouny, R., Smit, R.B., Zhao, T., Wong, R., Lee-Glover, L., Desrochers-Goyette, J., Ilamathi, H.S., Consortium, C.R.C et al. (2019) The R941L mutation in MYH14 disrupts mitochondrial fission and associates with peripheral neuropathy. EBioMedicine, 45, 379–392. 31231018 10.1016/j.ebiom.2019.06.018 PMC6642256

[32] Raudvere, U., Kolberg, L., Kuzmin, I., Arak, T., Adler, P., Peterson, H. and Vilo, J. (2019) g:Profiler: a web server for functional enrichment analysis and conversions of gene lists (2019 update). Nucleic Acids Res., 47, W191–W198. 31066453 10.1093/nar/gkz369 PMC6602461

[33] Supek, F., Bosnjak, M., Skunca, N. and Smuc, T. (2011) REVIGO summarizes and visualizes long lists of gene ontology terms. PLoS One, 6, e21800. 21789182 10.1371/journal.pone.0021800 PMC3138752

[34] Fisk, B., Ioannides, C.G., Aggarwal, S., Wharton, J.T., O'Brian, C.A., Restifo, N. and Glisson, B.S. (1994) Enhanced expression of HLA-A,B,C and inducibility of TAP-1, TAP-2, and HLA-A,B,C by interferon-gamma in a multidrug-resistant small cell lung cancer line. Lymphokine Cytokine Res., 13, 125–131. 8061113

[35] Subramanian, A., Tamayo, P., Mootha, V.K., Mukherjee, S., Ebert, B.L., Gillette, M.A., Paulovich, A., Pomeroy, S.L., Golub, T.R., Lander, E.S. and Mesirov, J.P. (2005) Gene set enrichment analysis: a knowledge-based approach for interpreting genome-wide expression profiles. Proc. Natl. Acad. Sci. U. S. A., 102, 15545–15550. 16199517 10.1073/pnas.0506580102 PMC1239896

[36] Liberzon, A., Subramanian, A., Pinchback, R., Thorvaldsdóttir, H., Tamayo, P. and Mesirov, J.P. (2011) Molecular signatures database (MSigDB) 3.0. Bioinformatics, 27, 1739–1740. 21546393 10.1093/bioinformatics/btr260 PMC3106198

[37] Miranda, S., Foncea, R., Guerrero, J. and Leighton, F. (1999) Oxidative stress and upregulation of mitochondrial biogenesis genes in mitochondrial DNA-depleted HeLa cells. Biochem. Biophys. Res. Commun., 258, 44–49. 10222232 10.1006/bbrc.1999.0580

[38] L'honore, A., Commere, P.-H., Negroni, E., Pallafacchina, G., Friguet, B., Drouin, J., Buckingham, M. and Montarras, D. (2018) The role of Pitx2 and Pitx3 in muscle stem cells gives new insights into P38a MAP kinase and redox regulation of muscle regeneration. elife, 7, e32991. 30106373 10.7554/eLife.32991 PMC6191287

[39] Chang, N.C., Nguyen, M. and Shore, G.C. (2012) BCL2-CISD2: an ER complex at the nexus of autophagy and calcium homeostasis? Autophagy, 8, 856–857. 22617439 10.4161/auto.20054

[40] Yeh, C.-H., Chou, Y.-J., Kao, C.-H. and Tsai, T.-F. (2020) Mitochondria and calcium homeostasis: Cisd2 as a big player in cardiac ageing. Int. J. Mol. Sci., 21, 9238. 33287440 10.3390/ijms21239238 PMC7731030

[41] Meinke, P., Schneiderat, P., Srsen, V., Korfali, N., Le Thanh, P., Cowan, G.J.M., Cavanagh, D.R., Wehnert, M., Schirmer, E.C. and Walter, M.C. (2015) Abnormal proliferation and spontaneous differentiation of myoblasts from a symptomatic female carrier of X-linked Emery-Dreifuss muscular dystrophy. Neuromuscul. Disord., 25, 127–136. 25454731 10.1016/j.nmd.2014.09.012 PMC4317192

[42] Jöbsis, G.J., Keizers, H., Vreijling, J.P., de Visser, M., Speer, M.C., Wolterman, R.A., Baas, F. and Bolhuis, P.A. (1996) Type VI collagen mutations in Bethlem myopathy, an autosomal dominant myopathy with contractures. Nat. Genet., 14, 113–115. 8782832 10.1038/ng0996-113

[43] Lampe, A.K., Dunn, D.M., von Niederhausern, A.C., Hamil, C., Aoyagi, A., Laval, S.H., Marie, S.K., Chu, M.-L., Swoboda, K., Muntoni, F. et al. (2005) Automated genomic sequence analysis of the three collagen VI genes: applications to Ullrich congenital muscular dystrophy and Bethlem myopathy. J. Med. Genet., 42, 108–120. 15689448 10.1136/jmg.2004.023754 PMC1736000

[44] Baker, N.L., Morgelin, M., Pace, R.A., Peat, R.A., Adams, N.E., McKinlay Gardner, R.J., Rowland, L.P., Miller, G., De Jonghe, P., Ceulemans, B. et al. (2007) Molecular consequences of dominant Bethlem myopathy collagen VI mutations. Ann. Neurol., 62, 390–405. 17886299 10.1002/ana.21213

[45] Zou, Y., Zwolanek, D., Izu, Y., Gandhy, S., Schreiber, G., Brockmann, K., Devoto, M., Tian, Z., Hu, Y., Veit, G. et al. (2014) Recessive and dominant mutations in COL12A1 cause a novel EDS/myopathy overlap syndrome in humans and mice. Hum. Mol. Genet., 23, 2339–2352. 24334604 10.1093/hmg/ddt627 PMC3976332

[46] Kaar, J.L., Li, Y., Blair, H.C., Asche, G., Koepsel, R.R., Huard, J. and Russell, A.J. (2008) Matrix metalloproteinase-1 treatment of muscle fibrosis. Acta Biomater., 4, 1141–1120. 10.1016/j.actbio.2008.03.010 18440885

[47] Zhu, X., Yao, T., Wang, R., Guo, S., Wang, X., Zhou, Z., Zhang, Y., Zhuo, X., Wang, R., Li, J.Z., Liu, T. and Kong, X. (2020) IRF4 in skeletal muscle regulates exercise capacity via PTG/glycogen pathway. Adv. Sci. (Weinh), 7, 2001502. 33042761 10.1002/advs.202001502 PMC7539189

[48] Chiba, K., Tsuchiya, M., Koide, M., Hagiwara, Y., Sasaki, K., Hattori, Y., Watanabe, M., Sugawara, S., Kanzaki, M. and Endo, Y. (2015) Involvement of IL-1 in the maintenance of masseter muscle activity and glucose homeostasis. PLoS One, 10, e0143635. 26599867 10.1371/journal.pone.0143635 PMC4658060

[49] Fairley, E., Riddell, A., Ellis, J. and Kendrick-Jones, J. (2002) The cell cycle dependent mislocalisation of emerin may contribute to the Emery-Dreifuss muscular dystrophy phenotype. J. Cell Sci., 115, 341–354. 11839786 10.1242/jcs.115.2.341

[50] Mademtzoglou, D., Asakura, Y., Borok, M.J., Alonso-Martin, S., Mourikis, P., Kodaka, Y., Mohan, A., Asakura, A. and Relaix, F. (2018) Cellular localization of the cell cycle inhibitor Cdkn1c controls growth arrest of adult skeletal muscle stem cells. elife, 7, e33337. 30284969 10.7554/eLife.33337 PMC6172026

[51] Caruelle, D., Mazouzi, Z., Husmann, I., Delbe, J., Duchesnay, A., Gautron, J., Martelly, I. and Courty, J. (2004) Upregulation of HARP during in vitro myogenesis and rat soleus muscle regeneration. J. Muscle Res. Cell Motil., 25, 45–53. 15160487 10.1023/b:jure.0000021387.92378.2e

[52] Dietrich, S., Abou-Rebyeh, F., Brohmann, H., Bladt, F., Sonnenberg-Riethmacher, E., Yamaai, T., Lumsden, A., Brand-Saberi, B. and Birchmeier, C. (1999) The role of SF/HGF and c-Met in the development of skeletal muscle. Development, 126, 1621–1629. 10079225 10.1242/dev.126.8.1621

[53] Mousavi, K. and Jasmin, B.J. (2006) BDNF is expressed in skeletal muscle satellite cells and inhibits myogenic differentiation. J. Neurosci., 26, 5739–5749. 16723531 10.1523/JNEUROSCI.5398-05.2006 PMC6675269

[54] Peng, H.B., Ali, A.A., Dai, Z., Daggett, D.F., Raulo, E. and Rauvala, H. (1995) The role of heparin-binding growth-associated molecule (HB-GAM) in the postsynaptic induction in cultured muscle cells. J. Neurosci., 15, 3027–3038. 7722643 10.1523/JNEUROSCI.15-04-03027.1995 PMC6577744

[55] Stark, D.A., Karvas, R.M., Siegel, A.L. and Cornelison, D.D. (2011) Eph/ephrin interactions modulate muscle satellite cell motility and patterning. Development, 138, 5279–5289. 22071104 10.1242/dev.068411 PMC3222207

[56] Singh, R., Artaza, J.N., Taylor, W.E., Gonzalez-Cadavid, N.F. and Bhasin, S. (2003) Androgens stimulate myogenic differentiation and inhibit adipogenesis in C3H 10T1/2 pluripotent cells through an androgen receptor-mediated pathway. Endocrinology, 144, 5081–5088. 12960001 10.1210/en.2003-0741

[57] Bryan, B.A., Mitchell, D.C., Zhao, L., Ma, W., Stafford, L.J., Teng, B.-B. and Liu, M. (2005) Modulation of muscle regeneration, myogenesis, and adipogenesis by the Rho family guanine nucleotide exchange factor GEFT. Mol. Cell. Biol., 25, 11089–11101. 16314529 10.1128/MCB.25.24.11089-11101.2005 PMC1316953

[58] Czapiewski, R., Batrakou, D.G., de Las Heras, J.I., Carter, R., Sivakumar, A., Sliwinska, M., Dixon, C.R., Webb, S., Lattanzi, G., Morton, N. et al. (2022) Genomic loci mispositioning in Tmem120a knockout mice yields latent lipodystrophy. Nat. Commun., 13, 321. 10.1038/s41467-41021-27869-41462. 35027552 PMC8758788

[59] Oldenburg, A.R., Delbarre, E., Thiede, B., Vigouroux, C. and Collas, P. (2014) Deregulation of Fragile X-related protein 1 by the lipodystrophic lamin A p.R482W mutation elicits a myogenic gene expression program in preadipocytes. Hum. Mol. Genet., 23, 1151–1162. 24108105 10.1093/hmg/ddt509

[60] Anczuków, O., Rosenberg, A.Z., Akerman, M., Das, S., Zhan, L., Karni, R., Muthuswamy, S.K. and Krainer, A.R. (2012) The splicing factor SRSF1 regulates apoptosis and proliferation to promote mammary epithelial cell transformation. Nat. Struct. Mol. Biol., 19, 220–228. 22245967 10.1038/nsmb.2207 PMC3272117

[61] Todorow, V., Hintze, S., Kerr, A.R.W., Hehr, A., Schoser, B. and Meinke, P. (2021) Transcriptome analysis in a primary human muscle cell differentiation model for myotonic dystrophy type 1. Int. J. Mol. Sci., 22, 8607. 34445314 10.3390/ijms22168607 PMC8395314

[62] Bachinski, L.L., Baggerly, K.A., Neubauer, V.L., Nixon, T.J., Raheem, O., Sirito, M., Unruh, A.K., Zhang, J., Nagarajan, L., Timchenko, L.T. et al. (2014) Most expression and splicing changes in myotonic dystrophy type 1 and type 2 skeletal muscle are shared with other muscular dystrophies. Neuromuscul. Disord., 24, 227–240. 24332166 10.1016/j.nmd.2013.11.001 PMC3943873

[63] Lee, K.-S., Cao, Y., Witwicka, H.E., Tom, S., Tapscott, S.J. and Wang, E.H. (2010) RNA-binding protein Muscleblind-like 3 (MBNL3) disrupts myocyte enhancer factor 2 (Mef2) {beta}-exon splicing. J. Biol. Chem., 285, 33779–33787. 20709755 10.1074/jbc.M110.124255 PMC2962477

[64] Hackman, P., Vihola, A., Haravuori, H., Marchand, S., Sarparanta, J., De Seze, J., Labeit, S., Witt, C., Peltonen, L., Richard, I. et al. (2002) Tibial muscular dystrophy is a titinopathy caused by mutations in TTN, the gene encoding the giant skeletal-muscle protein titin. Am. J. Hum. Genet., 71, 492–500. 12145747 10.1086/342380 PMC379188

[65] Penisson-Besnier, I., Hackman, P., Suominen, T., Sarparanta, J., Huovinen, S., Richard-Cremieux, I. and Udd, B. (2010) Myopathies caused by homozygous titin mutations: limb-girdle muscular dystrophy 2J and variations of phenotype. J. Neurol. Neurosurg. Psychiatry, 81, 1200–1202. 20571043 10.1136/jnnp.2009.178434

[66] Gundesli, H., Talim, B., Korkusuz, P., Balci-Hayta, B., Cirak, S., Akarsu, N.A., Topaloglu, H. and Dincer, P. (2010) Mutation in exon 1f of PLEC, leading to disruption of plectin isoform 1f, causes autosomal-recessive limb-girdle muscular dystrophy. Am. J. Hum. Genet., 87, 834–841. 21109228 10.1016/j.ajhg.2010.10.017 PMC2997373

[67] Richard, I., Broux, O., Allamand, V., Fougerousse, F., Chiannilkulchai, N., Bourg, N., Brenguier, L., Devaud, C., Pasturaud, P., Roudaut, C. et al. (1995) Mutations in the proteolytic enzyme calpain 3 cause limb-girdle muscular dystrophy type 2A. Cell, 81, 27–40. 7720071 10.1016/0092-8674(95)90368-2

[68] Vissing, J., Barresi, R., Witting, N., Van Ghelue, M., Gammelgaard, L., Bindoff, L.A., Straub, V., Lochmuller, H., Hudson, J., Wahl, C.M. et al. (2016) A heterozygous 21-bp deletion in CAPN3 causes dominantly inherited limb girdle muscular dystrophy. Brain, 139, 2154–2163. 27259757 10.1093/brain/aww133

[69] Roberds, S.L., Leturcq, F., Allamand, V., Piccolo, F., Jeanpierre, M., Anderson, R.D., Lim, L.E., Lee, J.C., Tome, F.M., Romero, N.B. et al. (1994) Missense mutations in the adhalin gene linked to autosomal recessive muscular dystrophy. Cell, 78, 625–633. 8069911 10.1016/0092-8674(94)90527-4

[70] Hoffman, E.P., Brown, R.H., Jr. and Kunkel, L.M. (1987) Dystrophin: the protein product of the Duchenne muscular dystrophy locus. Cell, 51, 919–928. 3319190 10.1016/0092-8674(87)90579-4

[71] Kunkel, L.M., Hejtmancik, J.F., Caskey, C.T., Speer, A., Monaco, A.P., Middlesworth, W., Colletti, C.A., Bertelson, C., Muller, U., Bresnan, M. et al. (1986) Analysis of deletions in DNA from patients with Becker and Duchenne muscular dystrophy. Nature, 322, 73–77. 3014348 10.1038/322073a0

[72] López-Martínez, A., Soblechero-Martín, P., de-la-Puente-Ovejero, L., Nogales-Gadea, G. and Arechavala-Gomeza, V. (2020) An overview of alternative splicing defects implicated in myotonic dystrophy type I. Genes, 11, 1109. 32971903 10.3390/genes11091109 PMC7564762

[73] Jia, Y., Jucius, T.J., Cook, S.A. and Ackerman, S.L. (2015) Loss of Clcc1 results in ER stress, misfolded protein accumulation, and neurodegeneration. J. Neurosci., 35, 3001–3009. 25698737 10.1523/JNEUROSCI.3678-14.2015 PMC4331624

[74] Isa, A., Nehlin, J.O., Sabir, H.J., Andersen, T.E., Gaster, M., Kassem, M. and Barington, T. (2010) Impaired cell surface expression of HLA-B antigens on mesenchymal stem cells and muscle cell progenitors. PLoS One, 5, e10900. 20531935 10.1371/journal.pone.0010900 PMC2878340

[75] O'Hanlon, T.P., Carrick, D.M., Targoff, I.N., Arnett, F.C., Reveille, J.D., Carrington, M., Gao, X., Oddis, C.V., Morel, P.A., Malley, J.D. et al. (2006) Immunogenetic risk and protective factors for the idiopathic inflammatory myopathies: distinct HLA-A, -B, -Cw, -DRB1, and -DQA1 allelic profiles distinguish European American patients with different myositis autoantibodies. Medicine, 85, 111–127. 16609350 10.1097/01.md.0000217525.82287.eb

[76] Melendez, J., Sieiro, D., Salgado, D., Morin, V., Dejardin, M.-J., Zhou, C., Mullen, A.C. and Marcelle, C. (2021) TGFβ signalling acts as a molecular brake of myoblast fusion. Nat. Commun., 12, 749. 33531476 10.1038/s41467-020-20290-1 PMC7854724

[77] Yue, L., Wentao, L., Xin, Z., Jingjing, H., Xiaoyan, Z., Na, F., Tonghui, M. and Dalin, L. (2020) Human epidermal growth factor receptor 2-positive metastatic breast cancer with novel epidermal growth factor receptor -ZNF880 fusion and epidermal growth factor receptor E114K mutations effectively treated with pyrotinib: a case report. Medicine (Baltimore), 99, e23406. 33371069 10.1097/MD.0000000000023406 PMC7748359

[78] Bala, P., Singh, A.K., Kavadipula, P., Kotapalli, V., Sabarinathan, R. and Bashyam, M.D. (2021) Exome sequencing identifies ARID2 as a novel tumor suppressor in early-onset sporadic rectal cancer. Oncogene, 40, 863–874. 33262464 10.1038/s41388-020-01537-z

[79] Cacchiarelli, D., Legnini, I., Martone, J., Cazzella, V., D'Amico, A., Bertini, E. and Bozzoni, I. (2011) miRNAs as serum biomarkers for Duchenne muscular dystrophy. EMBO Mol. Med., 3, 258–265. 21425469 10.1002/emmm.201100133 PMC3112257

[80] Israeli, D., Poupiot, J., Amor, F., Charton, K., Lostal, W., Jeanson-Leh, L. and Richard, I. (2016) Circulating miRNAs are generic and versatile therapeutic monitoring biomarkers in muscular dystrophies. Sci. Rep., 6, 28097. 27323895 10.1038/srep28097 PMC4914855

[81] Sylvius, N., Bonne, G., Straatman, K., Reddy, T., Gant, T.W. and Shackleton, S. (2011) MicroRNA expression profiling in patients with lamin A/C-associated muscular dystrophy. FASEB J., 25, 3966–3978. 21840938 10.1096/fj.11-182915

[82] Fedak, P.W.M., Moravee, C.S., McCarthy, P.M., Altamentova, S.M., Wong, A.P., Skrtic, M., Verma, S., Weisel, R.D. and Li, R.-K. (2006) Altered expression of disintegrin metalloproteinases and their inhibitor in human dilated cardiomyopathy. Circulation, 113, 238–245. 16401770 10.1161/CIRCULATIONAHA.105.571414

[83] Winckler, P.B., da Silva, A.M.S., Coimbra-Neto, A.R., Carvalho, E., Cavalcanti, E.B.U., Sobreira, C.F.R., Marrone, C.D., Machado-Costa, M.C., Carvalho, A.A.S., Feio, R.H.F. et al. (2019) Clinicogenetic lessons from 370 patients with autosomal recessive limb-girdle muscular dystrophy. Clin. Genet., 96, 341–353. 31268554 10.1111/cge.13597

[84] Bernasconi, P., Carboni, N., Ricci, G., Siciliano, G., Politano, L., Maggi, L., Mongini, T., Vercelli, L., Rodolico, C., Biagini, E. et al. (2018) Elevated TGF β2 serum levels in Emery-Dreifuss muscular dystrophy: implications for myocyte and tenocyte differentiation and fibrogenic processes. Nucleus, 9, 292–304. 29693488 10.1080/19491034.2018.1467722 PMC5973167

[85] Lehka, L. and Redowicz, M.J. (2020) Mechanisms regulating myoblast fusion: a multilevel interplay. Semin. Cell Dev. Biol., 104, 81–92. 32063453 10.1016/j.semcdb.2020.02.004

[86] Fan, S., Tian, T., Chen, W., Lv, X., Lei, X., Zhang, H., Sun, S., Cai, L., Pan, G., He, L. et al. (2019) Mitochondrial miRNA determines chemoresistance by reprogramming metabolism and regulating mitochondrial transcription. Cancer Res., 79, 1069–1084. 30659020 10.1158/0008-5472.CAN-18-2505 PMC8631248

[87] Narozna, M. and Rubis, B. (2021) Anti-SARS-CoV-2 strategies and the potential role of miRNA in the assessment of COVID-19 morbidity, recurrence, and therapy. Int. J. Mol. Sci., 22, 8663. 34445368 10.3390/ijms22168663 PMC8395427

[88] Liao, W., Liang, P., Liu, B., Xu, Z., Zhang, L., Feng, M., Tang, Y. and Xu, A. (2020) MicroRNA-140-5p mediates renal fibrosis through TGF-β1/Smad signaling pathway by directly targeting TGFBR1. Front. Physiol., 11, 1093. 33013464 10.3389/fphys.2020.01093 PMC7499471

[89] Zhang, X., Chang, A., Li, Y., Gao, Y., Wang, H., Ma, Z., Li, X. and Wang, B. (2015) miR-140-5p regulates adipocyte differentiation by targeting transforming growth factor-β signaling. Sci. Rep., 5, 18118. 26657345 10.1038/srep18118 PMC4676041

[90] Liu, L., Li, T.-M., Liu, X.-R., Bai, Y.-P., Li, J., Tang, N. and Wang, X.-B. (2019) MicroRNA-140 inhibits skeletal muscle glycolysis and atrophy in endotoxin-induced sepsis in mice via the WNT signaling pathway. Am. J. Physiol. Cell Physiol., 317, C189–C199. 31042421 10.1152/ajpcell.00419.2018

[91] Feng, B., Chen, S., Gordon, A.D. and Chakrabarti, S. (2017) miR-146a mediates inflammatory changes and fibrosis in the heart in diabetes. J. Mol. Cell. Cardiol., 105, 70–76. 28279663 10.1016/j.yjmcc.2017.03.002

[92] Liu, Y., Luo, Y., Shen, L., Guo, R., Zhan, Z., Yuan, N., Sha, R., Qian, W., Wang, Z., Xie, Z., Wu, W. and Feng, Y. (2020) Splicing factor SRSF1 is essential for satellite cell proliferation and postnatal maturation of neuromuscular junctions in mice. Stem Cell Rep., 15, 941–954. 10.1016/j.stemcr.2020.08.004 PMC7561493 32888503

[93] Zullo, J.M., Demarco, I.A., Pique-Regi, R., Gaffney, D.J., Epstein, C.B., Spooner, C.J., Luperchio, T.R., Bernstein, B.E., Pritchard, J.K., Reddy, K.L. et al. (2012) DNA sequence-dependent compartmentalization and silencing of chromatin at the nuclear lamina. Cell, 149, 1474–1487. 22726435 10.1016/j.cell.2012.04.035

[94] Demmerle, J., Koch, A.J. and Holaska, J.M. (2013) Emerin and histone deacetylase 3 (HDAC3) cooperatively regulate expression and nuclear positions of MyoD, Myf5, and Pax7 genes during myogenesis. Chromosom. Res., 21, 765–779. 10.1007/s10577-013-9381-9 PMC4829207 24062260

[95] Zwerger, M., Jaalouk, D.E., Lombardi, M.L., Isermann, P., Mauermann, M., Dialynas, G., Herrmann, H., Wallrath, L.L. and Lammerding, J. (2013) Myopathic lamin mutations impair nuclear stability in cells and tissue and disrupt nucleo-cytoskeletal coupling. Hum. Mol. Genet., 22, 2335–2349. 23427149 10.1093/hmg/ddt079 PMC3658163

[96] Ziat, E., Mamchaoui, K., Beuvin, M., Nelson, I., Azibani, F., Spuler, S., Bonne, G. and Bertrand, A.T. (2016) FHL1B interacts with Lamin A/C and Emerin at the nuclear lamina and is Misregulated in Emery-Dreifuss muscular dystrophy. Neuromuscul. Disord., 3, 497–510. 10.3233/JND-160169 27911330

[97] Bonne, G., Leturcq, F. and Yaou, R.B. (2004) Emery-Dreifuss muscular dystrophy. GeneReviews, PMID:20301609. https://www.ncbi.nlm.nih.gov/books/NBK1436/.

[98] Dobin, A., Davis, C.A., Schlesinger, F., Drenkow, J., Zaleski, C., Jha, S., Batut, P., Chaisson, M. and Gingeras, T.R. (2013) STAR: ultrafast universal RNA-seq aligner. Bioinformatics, 29, 15–21. 23104886 10.1093/bioinformatics/bts635 PMC3530905

[99] Bolger, A.M., Lohse, M. and Usadel, B. (2014) Trimmomatic: a flexible trimmer for Illumina sequence data. Bioinformatics, 30, 2114–2120. 24695404 10.1093/bioinformatics/btu170 PMC4103590

[100] Love, M.I., HUber, W. and Anders, S. (2014) Moderated estimation of fold change and dispersion for RNA-seq data with DESeq2. Genome Biol., 15, 550. 25516281 10.1186/s13059-014-0550-8 PMC4302049

[101] Patro, R., Duggal, G., Love, M.I., Irizarry, R.A. and Kingsford, C. (2017) Salmon: fast and bias-aware quantification of transcript expression using dual-phase inference. Nat. Methods, 14, 417–419. 28263959 10.1038/nmeth.4197 PMC5600148

[102] Ritchie, M.E., Phipson, B., Wu, D., Hu, Y., Law, C.W., Shi, W. and Smyth, G.K. (2015) Limma powers differential expression analyses for RNA-sequencing and microarray studies. Nucleic Acids Res., 43, e47. 25605792 10.1093/nar/gkv007 PMC4402510

[103] Reimand, J., Arak, T., Adler, P., Kolberg, L., Reisberg, S., Peterson, H. and Vilo, J. (2016) g:Profiler-a web server for functional interpretation of gene lists (2016 update). Nucleic Acids Res., 44, W83–W89. 27098042 10.1093/nar/gkw199 PMC4987867

[104] Jain, A. and Tuteja, G. (2019) TissueEnrich: tissue-specific gene enrichment analysis. Bioinformatics, 35, 1966–1967. 30346488 10.1093/bioinformatics/bty890 PMC6546155

[105] Shannon, P., Markiel, A., Ozier, O., Baliga, N.S., Wang, J.T., Ramage, D., Amin, N., Schwikowski, B. and Ideker, T. (2003) Cytoscape: a software environment for integrated models of biomolecular interaction networks. Genome Res., 13, 2498–2504. 14597658 10.1101/gr.1239303 PMC403769

[106] Rooney, J.P., Ryde, I.T., Sanders, L.H., Howlett, E.H., Colton, M.D., Germ, K.E., Mayer, G.D., Greenamyre, J.T. and Meyer, J.N. (2015) PCR based determination of mitochondrial DNA copy number in multiple species. Methods Mol. Biol., 1241, 23–38. 25308485 10.1007/978-1-4939-1875-1_3 PMC4312664

[107] Nakhle, J., Ozkan, T., Lněničková, K., Briolotti, P. and Vignais, M.-L. (2020) Methods for simultaneous and quantitative isolation of mitochondrial DNA, nuclear DNA and RNA from mammalian cells. BioTechniques, 69, 436–442. 33103926 10.2144/btn-2020-0114

[108] Anders, S., Reyes, A. and Huber, W. (2012) Detecting differential usage of exons from RNA-seq data. Genome Res., 22, 2008–2017. 22722343 10.1101/gr.133744.111 PMC3460195

[109] Shen, S., Park, J.W., Lu, Z.-X. and Xing, Y. (2014) rMATS: robust and flexible detection of differential alternative splicing from replicate RNA-Seq data. Proc. Natl. Acad. Sci. U. S. A., 111, E5593–E5601. 25480548 10.1073/pnas.1419161111 PMC4280593

[110] Bray, N.L., Pimentel, H., Melsted, P. and Pachter, L. (2016) Near-optimal probabilistic RNA-seq quantification. Nat. Biotechnol., 34, 525–527. 27043002 10.1038/nbt.3519

[111] Vitting-Seerup, K. and Sandelin, A. (2019) IsoformSwitchAnalyzeR: analysis of changes in genome-wide patterns of alternative splicing and its functional consequences. Bioinformatics, 35, 4469–4471. 30989184 10.1093/bioinformatics/btz247

[112] Walter, W., Sánchez-Cabo, F. and Ricote, M. (2015) GOplot: an R package for visually combining expression data with functional analysis. Bioinformatics, 31, 2912–2914. 25964631 10.1093/bioinformatics/btv300

[113] Kolberg, L., Raudvere, U., Kuzmin, I., Vilo, J. and Peterson, H. (2020) gprofiler2 -- an R package for gene list functional enrichment analysis and namespace conversion toolset g:Profiler. F1000Res, 9, ELIXIR-709. 10.12688/f1000research.24956.1 PMC7859841 33564394

[114] Kang, Y.-J., Yang, D.-C., Kong, L., Hou, M., Meng, Y.-Q., Wei, L. and Gao, G. (2017) CPC2: a fast and accurate coding potential calculator based on sequence intrinsic features. Nucleic Acids Res., 45, W12–W16. 28521017 10.1093/nar/gkx428 PMC5793834

[115] Finn, R.D., Clements, J. and Eddy, S.R. (2011) HMMER web server: interactive sequence similarity searching. Nucleic Acids Res., 39, W29–W37. 21593126 10.1093/nar/gkr367 PMC3125773

[116] Nielsen, H., Engelbrecht, J., Brunak, S. and von Heijne, G. (1997) A neural network method for identification of prokaryotic and eukaryotic signal peptides and prediction of their cleavage sites. Int. J. Neural Syst., 8, 581–599. 10065837 10.1142/s0129065797000537

[117] Meszaros, B., Erdos, G. and Dosztányi, Z. (2018) IUPred2A: context-dependent prediction of protein disorder as a function of redox state and protein binding. Nucleic Acids Res., 46, W329–W337. 29860432 10.1093/nar/gky384 PMC6030935

